# Long periodic ripple in a 2D hybrid halide perovskite structure using branched organic spacers†

**DOI:** 10.1039/d0sc04144k

**Published:** 2020-10-06

**Authors:** Justin M. Hoffman, Christos D. Malliakas, Siraj Sidhik, Ido Hadar, Rebecca McClain, Aditya D. Mohite, Mercouri G. Kanatzidis

**Affiliations:** Department of Chemistry, Northwestern University Evanston IL 60208 USA m-kanatzidis@northwestern.edu; Department of Chemical and Biomolecular Engineering, Rice University Houston Texas 77005 USA

## Abstract

Two-dimensional (2D) halide perovskites have great promise in optoelectronic devices because of their stability and optical tunability, but the subtle effects on the inorganic layer when modifying the organic spacer remain unclear. Here, we introduce two homologous series of Ruddlesden–Popper (RP) structures using the branched isobutylammonium (IBA) and isoamylammonium (IAA) cations with the general formula (RA)_2_(MA)_*n*−1_Pb_*n*_I_3*n*+1_ (RA = IBA, IAA; MA = methylammonium *n* = 1–4). Surprisingly, the IAA *n* = 2 member results in the first modulated 2D perovskite structure with a ripple with a periodicity of 50.6 Å occurring in the inorganic slab diagonally to the [101] direction of the basic unit cell. This leads to an increase of Pb–I–Pb angles along the direction of the wave. Generally, both series show larger in-plane bond angles resulting from the additional bulkiness of the spacers compensating for the MA's small size. Larger bond angles have been shown to decrease the bandgap which is seen here with the bulkier IBA leading to both larger in-plane angles and lower bandgaps except for *n* = 2, in which the modulated structure has a lower bandgap because of its larger Pb–I–Pb angles. Photo-response was tested for the *n* = 4 compounds and confirmed, signaling their potential use in solar cell devices. We made films using an MACl additive which showed good crystallinity and preferred orientation according to grazing-incidence wide-angle scattering (GIWAXS). As exemplar, the two *n* = 4 samples were employed in devices with champion efficiencies of 8.22% and 7.32% for IBA and IAA, respectively.

## Abstract 2

Using branched spacer cations results in a modulated rippled 2D perovskite structure and materials for solar cells.

## Graphical abstract

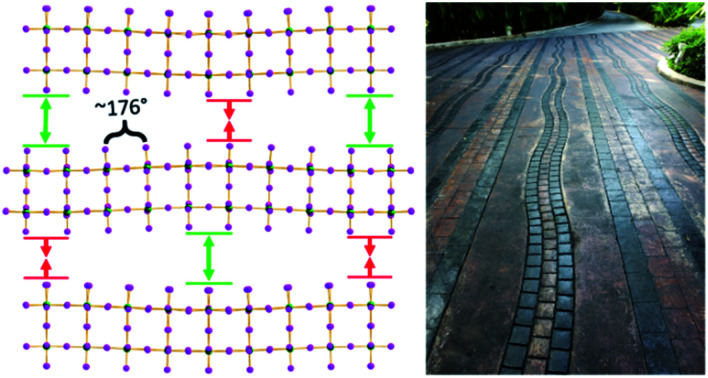

## Introduction

Hybrid halide perovskites have shown tremendous promise in photovoltaic devices with record efficiencies over 25% for optimized single junction solar cell devices.^1–8^ Though the materials used for these devices have intrinsic stability issues, using two-dimensional (2D) halide perovskites leads to greater long-term stability.^9–15^ Despite this, power conversion efficiencies using these materials are still lower, with few instances above 13% PCE for pure 2D materials.^16,17^ Using 2D/3D composite heterostructures has allowed higher efficiencies of over 20% with proper cation choice, but still not on par with the 3D materials, emphasizing the need for more choices of 2D materials for optimization of photovoltaic devices.^18,19^

Halide perovskites are a highly studied class of materials with the formula AMX_3_, in which A is a small cation, M is a metal, and X is a halide. The structure is composed of MX_6_ octahedra connected at the corners with the A cations sitting in cages created by the corner-sharing octahedra. In the field of photovoltaics, the best materials for high-efficiency solar cells have A as MA^+^ (methylammonium), FA^+^ (formamidinium), or Cs^+^; M as Pb^2+^ or Sn^2+^; and X as I^−^, Cl^−^, or Br^−^. MAPbI_3_ gives some of the highest efficiencies of the possible materials, partly because its bandgap of 1.52 eV (ref. 20) is close to the Shockley–Queisser ideal bandgap for single-junction solar cells of 1.34 eV.^21^ The bandgap can be further tuned by mixing the A-site cation,^22–24^ the metal,^25–27^ and the halide,^28,29^ leading to higher efficiencies,^30^ but mixing the halides leads to phase segregation, hindering the applicability of the devices.^31^ Meanwhile, mixing the A-site cations leads to changes in bond angles in the structure, which leads to a lower bandgap as the angles near 180°.^32^ The effects of the cations on the structure can be used to find the ideal bandgap for devices.

In 2D perovskites, the formula becomes 
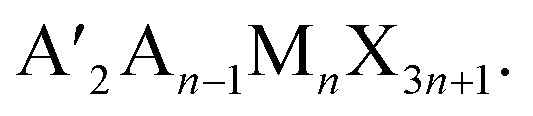
 In this case, a second cation, A′, has been added, which is too large to fit in the octahedron cages, leading to a reduced dimensionality structure composed of 2D sheets of corner-sharing octahedra with a thickness *n*.^33^ The 2D materials exhibit quantum and dielectric confinement which leads to a wider bandgap. Increasing the layer thickness *n* reduces the confinement, narrowing the bandgap, allowing for a large range of potential bandgaps based on *n*.^34–36^ The bandgap can further be tuned based on which A′ cation is used in the structure, like the mixed cation approach in 3D structures. For the 2D structures, a larger variety of ammonium cations can be used, allowing for an enormous degree of structural and property tunability. While there have been many studies of the *n* = 1 materials using different cations and their effects on bandgap,^37–42^ fewer studies have been performed on *n* > 1 materials. Furthermore, while it is well-established that the bandgap is determined heavily by the Pb–I–Pb bond angle,^15,37^ the correlation between bond angle and cation is less understood, particularly in structures of *n* > 1. Recently, we have reported the effects of using straight chain alkylamines of varying lengths on structure and properties in the homologous series (CH_3_(CH_2_)_*m*_NH_3_)_2_(CH_3_NH_3_)_*n*−1_Pb_*n*_I_3*n*+1_ (*m* = 2, *n* = 3, 4; *m* = 3, *n* = 2–7; *m* = 4, *n* = 2–5; *m* = 5, *n* = 2–4).^12,43^ We have also reported perovskites employing straight chain alkyldiamines (NH_3_C_*m*_H_2*m*_NH_3_)(CH_3_NH_3_)_*n*−1_Pb_*n*_I_3*n*+1_ (*m* = 4–9; *n* = 1–4) of varying lengths.^13^ While other cations have stabilized a variety of 2D structures such as Dion–Jacobson (DJ) perovskites^15,44,45^ and alternating cations in the interlayer space (ACI)^14,46^ amongst others,^33,47,48^ the cations used to stabilize such structures deviate greatly from the alkylamines. By looking at small changes in the cations, trends in structure, stability, and properties can be more rationally understood.

In order to further tune the structure and draw correlations between the cations and the resulting 2D perovskite structure, we employed the branch ammonium cations isobutylammonium (IBA) and isoamylammonium (IAA) in the synthesis of 2D perovskite structures with *n* = 1–4. IBA has already shown promise in solar cell devices because of its increased rate of charge transfer,^49,50^ making the effect of bulky branched spacers on the structure and properties an important point to understand. IBA and IAA are approximately the same length as the straight chain alkylamines propylamine (PA) and butylamine (BA), respectively, allowing for comparisons to these less bulky equivalents. Eight compounds with the formula (RA)_2_(MA)Pb_*n*_I_3*n*+1_ (RA = IBA, IAA; *n* = 1–4) were synthesized using a solution synthesis. We report that the IAA *n* = 2 is the first discovered modulated 2D perovskite structure, with the layers exhibiting curvature and distorting in a long-range wave with a periodicity of eight PbI_6_ units. This new modulated structure leads to increased Pb–I–Pb angles in the structure along the direction of the wave modulation as well as a lower bandgap. In general, we found that the branch amine series lead to larger angles when compared to the straight-chain alkylamines of the same length, which is attributed to the bulkiness of the branched spacers. Along with this, current–voltage mobility (*I*–*V*) measurements showed good conductivity along the layers and a photoresponse, leading us to test the *n* = 4 structures in films and solar cell devices. Using a MACl additive, films with good crystallinity were fabricated and made into solar cells with a champion efficiency of 8.22%, showing that these are excellent candidates for photovoltaic applications.

## Results and discussion

### Synthesis and structural properties

**Fig. 1:**
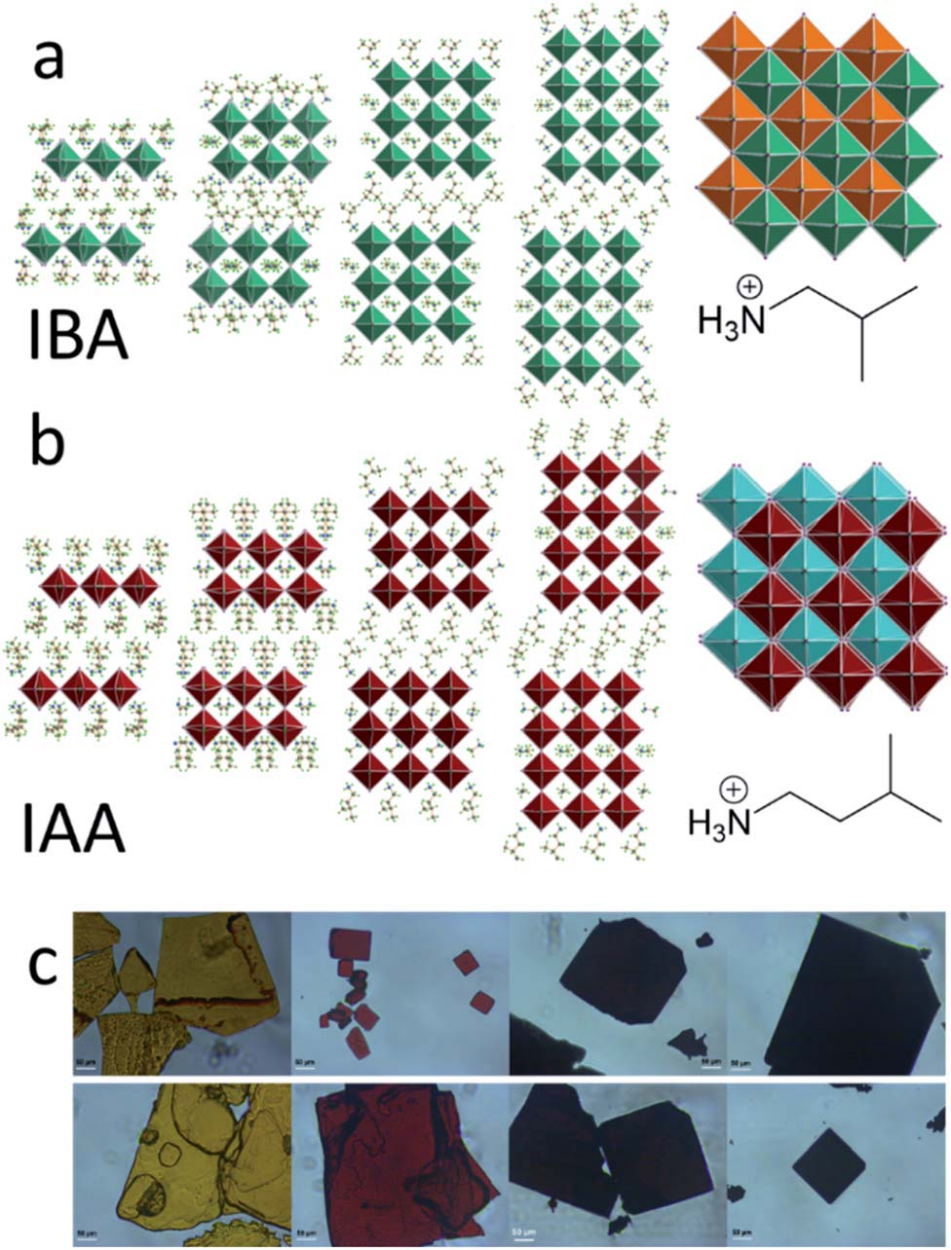
(a and b) Crystal structures of *n* = 1–4 for (a) IBA and (b) IAA. The structure for IAA *n* = 2 is shown without modulation. On the right is the view of the structures perpendicular to the layers of *n* = 3 for IBA and IAA showing the similar in-plane distortion of the octahedra. Different layers are shown in different colors. (c) Optical images of crystals of IBA (top) and IAA (bottom) in order of increase n from left to right.

The synthetic methods used here were like those of the previously reported multilayered 2D halide perovskites.^12–15,34,43^ PbO and MACl were simultaneously dissolved in hydriodic acid (HI) and hydrophosphorous acid (H_3_PO_2_), to give a yellow solution which was heated to a boil. Separately, a half-stoichiometric ratio of the spacer (IAA or IBA) was slowly added in HI in an ice bath, as the reaction is very exothermic. This solution was then added to the boiling solution. For IAA, the resulting iodide salt is less soluble at low temperatures, leading to the formation of a white precipitate of (IAA)I in the HI during neutralization. Fortunately, the precipitation of this salt leads to a noticeably less exothermic neutralization process. This precipitate was re-dissolved by heating the neutralized solution briefly at 100 °C before adding it to the boiling solution. Once all reagents were added, the yellow solution was cooled naturally on the hotplate, leading to better crystal quality and phase purity.^12^ Upon cooling and crystallization (2–3 hours), the crystals were dried to prevent disproportionation of different *n* values which occurs slowly while the crystals sit in the HI solution.^43^ The resulting crystals are plate-like and generally have a rectangular shape. However, the IAA *n* = 1 crystals form as irregularly shaped plates. In both cases, the *n* = 1 crystals were orange, *n* = 2 red, *n* = 3 dark red, and *n* = 4 black (Fig. 1c). The resulting crystals were then measured by single-crystal X-ray diffraction to refine their crystal structure.

Crystal structures are shown in Fig. 1 with relevant crystallographic information available in Table 1 with relevant angles in Tables S1–S24.† All structures were solved in the monoclinic system. In the IBA series, the structures for *n* = 1 and *n* = 2 have been previously solved as *P*2_1_/*c* and *Cc*, respectively.^51^ Here, we resolved *n* = 2 as *C*2/*c*, as the highly symmetric branched cations allowed the structure to be solved and refined in the centrosymmetric space group. We also successfully refined the *n* = 3 and *n* = 4 structures in the *C*2/*c* space group. For the IAA series, *n* = 1 was also refined as *P*2_1_/*c*, but *n* = 3 and 4 were solved as *C*2/*c* and *C*2/*m*. For structures of *n* = 2 and higher, the unit cell is also doubled along the long axis for all structures, similar to the BA and GA series,^14,34^ but a much larger supercell was found, which will be discussed below. All structures can be defined as Ruddlesden–Popper (RP) type in which the layers are shifted by 1/2 of a unit cell along one of the crystallographic axes parallel to the layers (Fig. 1a and b).

Crystal data and structure refinement for IAA and IBA compounds^a^

**Table:** 

	(IBA)_2_PbI_4_	(IBA)_2_(MA)Pb_2_I_7_	(IBA)_2_(MA)_2_Pb_3_I_10_	(IBA)_2_(MA)_3_Pb_4_I_13_
Crystal system	Monoclinic	Monoclinic	Monoclinic	Monoclinic
Space group	*P*2_1_/*c*	*C*2/*c*	*C*2/*c*	*C*2/*c*
Unit cell dimensions (Å)	13.8298(7)	38.9330(15)	8.9429(6)	8.9397(6)
	8.9872(3)	8.9419(2)	8.9415(4)	8.9386(7)
	8.7822(3)	8.8520(3)	51.048(3)	63.648(4)
	108.213(5)°	90.112(3)°	90.006(5)°	90.015(6)°
Volume (Å^3^)	1036.86(8)	3081.69(17)	4082.0(16)	5086(2)
*Z*	2	4	4	4
Density (calcd) (g cm^−3^)	2.7645	3.1966	3.4416	3.5562
Absorption coefficient	14.074	17.924	19.915	21.104
Independent reflections	12 785 [*R*_int_ = 0.050]	2768 [*R*_int_ = 0.0387]	3587 [*R*_int_ = 0.2104]	6873 [*R*_int_ = 0.3699]
Completeness	99%	98%	97%	94%
Data/restraints/parameters	12 785/8/41	9867/9/62	3587/9/84	6873/10/105
Goodness-of-fit	2.40	5.41	3.57	1.30
Final *R* indices [*I* > 2*σ*(*I*)]	*R*_obs_ = 0.0342	*R*_obs_ = 0.0667	*R*_obs_ = 0.1190	*R*_obs_ = 0.0980
	w*R*_obs_ = 0.0911	w*R*_obs_ = 0.2027	w*R*_obs_ = 0.2492	w*R*_obs_ = 0.1804
*R* indices [all data]	*R*_all_ = 0.0366	*R*_all_ = 0.0730	*R*_all_ = 0.2317	*R*_all_ = 0.4058
	w*R*_all_ = 0.0917	w*R*_all_ = 0.2033	w*R*_all_ = 0.2606	w*R*_all_ = 0.2094
Largest diff. peak and hole (e·Å^3^)	1.61 and −1.60	3.12 and −2.58	3.98 and −4.84	4.45 and −2.41

a *R* = ∑‖*F*_o_| − |*F*_c_‖/∑|*F*_o_|, w*R* = {∑[w(|*F*_o_|^2^ − |*F*_c_|^2^)^2^]/∑[w(|*F*_o_|^4^)]}^1/2^ and w = 1/(*σ*^2^(*I*) + 0.0004*I*^2^).

**Table:** 

	(IAA)_2_PbI_4_	(IAA)_2_(MA)Pb_2_I_7_	(IAA)_2_(MA)_2_Pb_3_I_10_	(IAA)_2_(MA)_3_Pb_4_I_13_
Crystal system	Monoclinic	Monoclinic	Monoclinic	Monoclinic
Space group	*P*2_1_/*c*	*P*2(*α*0*γ*)0	*C*2/*c*	*C*2/*m*
Unit cell dimensions (Å)	16.559(4)	8.8957(4)	8.9061(4)	8.9275(10)
	8.7623(14)	42.8038(19)	55.689(3)	68.140(10)
	8.7693(16)	8.9021(4)	8.9027(5)	8.9142(10)
	105.3538(16)°	89.326(3)°	90.055(4)°	90.551(9)°
*q*-Vector (1)	N/A	1/8a* + 1/8c*	N/A	N/A
Volume (Å^3^)	1227.0(5)	3389.4(3)	4415.5(16)	5422.4(12)
*Z*	2	4	4	4
Density (calcd) (g cm^−3^)	2.4121	2.5764	3.2058	3.3699
Absorption coefficient (mm^−1^)	11.898	16.277	18.412	19.797
Independent reflections	3105 [*R*_int_ = 0.1924]	7455 (2766 main + 5029 satellites) [*R*_int_ = 0.07]	4003 [*R*_int_ = 0.1334]	4785 [*R*_int_ = 0.3061]
Completeness	99%	97%	98%	97%
Data/restraints/parameters	3105/10/46	7455/0/496	4003/11/90	4785/12/114
Goodness-of-fit	3.30	2.25	2.32	0.80
Final *R* indices [*I* > 2*σ*(*I*)]	*R*_obs_ = 0.0757	*R*_obs_ = 0.0963	*R*_obs_ = 0.0997	*R*_obs_ = 0.0951
	w*R*_obs_ = 0.1835	w*R*_obs_ = 0.1737	w*R*_obs_ = 0.1892	w*R*_obs_ = 0.1971
*R* indices [all data]	*R*_all_ = 0.1811	*R*_all_ = 0.2302	*R*_all_ = 0.2075	*R*_all_ = 0.3343
	w*R*_all_ = 0.1967	w*R*_all_ = 0.1895	w*R*_all_ = 0.1958	w*R*_all_ = 0.2309
Largest diff. peak and hole (e·Å^−3^)	2.78 and −2.07	2.70 and −2.97	5.91 and −7.14	1.95 and −2.56

**Fig. 2:**
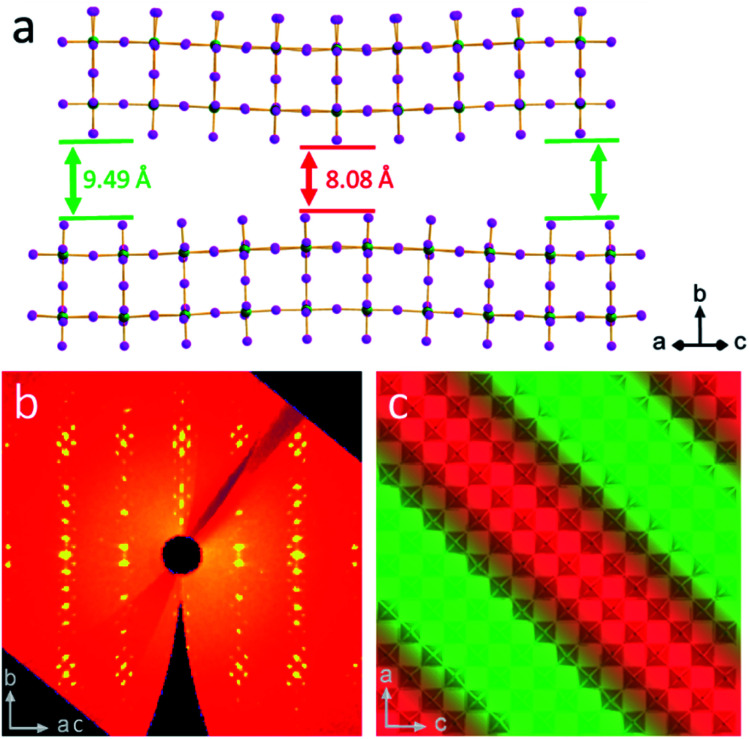
(a) The modulated IAA *n* = 2 structure viewed along the (1 0 1) axis showing a single wave in the structure. The minimum and maximum interlayer distances are labeled in red and green, respectively. (b) The diffraction of a single crystal showing the supercell reflections. (c) A view of the crystal structure looking perpendicular to a single layer. The areas colored in green indicate maximum interlayer spacing between two arbitrary layers, and red indicates minimum interlayer spacing.

For the IAA *n* = 2 member, a very large supercell was found, the first of its type identified in 2D halide perovskites. The supercell was evident from the satellite Bragg reflections seen looking down the *c*-axis of the crystals (Fig. 2b). The structure is therefore modulated and was best solved using the four-dimensional space group *P*2(*α*0*γ*)0 with a unit cell of *a* = 8.8957(4) Å, *b* = 42.8038(19) Å, *c* = 8.9021(4) Å, *β* = 90.674(3)° and a diagonal modulation vector *q* = 1/8a* + 1/8c*. This results in one wave diagonal to the unit cell, which is seen physically in the structure as a ripple along the chains of Pb–I bonds in the structure (Fig. 2a). This ripple gives rise to the 1/8a* − 1/8c* *q*-vector which is an 8 × 8 supercell and thus repeats every eight octahedra along the *a* and *c* axes with an amplitude of 0.36 Å. This leads to a substantial difference of 1.41 Å between the minimum and maximum interlayer spacing. Fig. 2c shows a panoramic view of the perovskite layers in the structure to highlight the direction of the wave, with the minimum interlayer spacing in highlighted red and maximum in green. The effects of this wave on bond angles and interlayer spacing will be discussed further below. While it is unclear why this is the only one of the eight structures reported to have such a modulation, it is likely that the reasons for this are because of a complex interplay of the steric effects of the MA and IAA cations and the van der Waals forces between the layers which is too complex to be addressed without further theoretical studies.

**Fig. 3:**
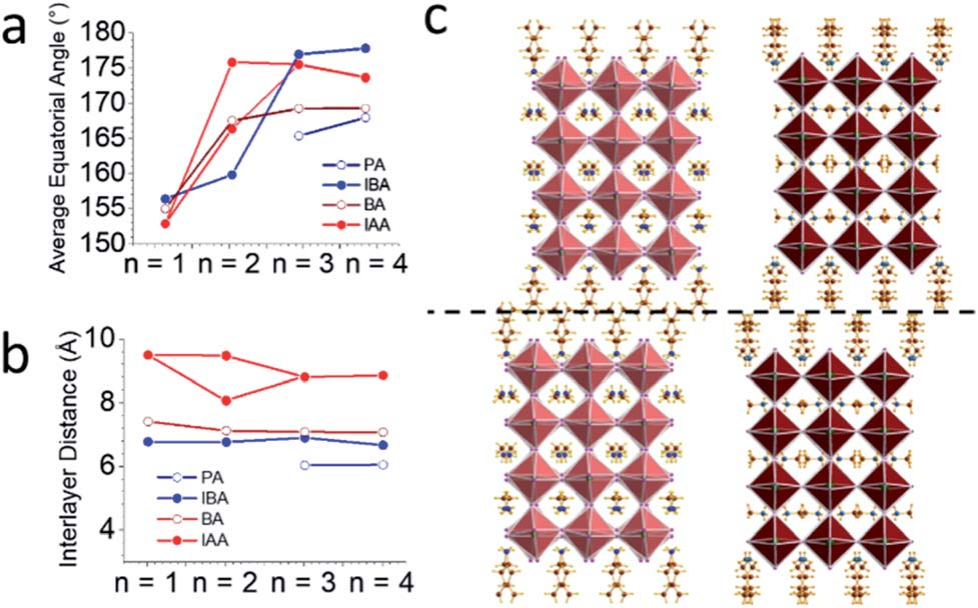
(a) The average equatorial angles for branched and straight-chain amines shows the overall increase in angle for the branched amines. For IAA *n* = 2, two points are shown. The top is for the direction along the wave modulation and the bottom for the direction perpendicular to it. (b) The interlayer spacing for branched and straight-chain amines shows the increase in spacing for the branched amines. Note that for IAA *n* = 2, both the minimum and maximum interlayer spacings were plotted here. (c) A comparison of interlayer spacing for BA (left) and IAA (right) with the bold line in the center highlighting the inability of the branch amines to cross.

The bulkiness of the spacer compared to the straight chain alkylamine spacers has an unanticipated influence on the distortions of the PbI_6_ octahedra in all the structures. While it may be expected that the branched spacers would require greater distortion to accommodate their bulkiness, the overall trend shows the opposite. To characterize the distortion of the octahedra, the Pb–I–Pb angles were calculated in two directions: out-of-plane to the layers (the axial angles) and in-plane to the layers (the equatorial angles). Fig. 1a and b shows that the octahedra tend to tilt more in-plane than out-of-plane, particularly when *n* is low. The axial angles are closer to 180° than for the branched spacers, particularly IBA, when compared to the straight chain amines. The equatorial angles are more distorted than the axial angles, but the equatorial angles approach 180° as *n* increases (Fig. 3a). In this direction, the branched amines show larger Pb–I–Pb angles for higher *n* structures compared to the straight-chain amines even though the trend is less obvious for *n* < 3.^34,43^ This decrease in distortion for higher *n* has implications for photovoltaics, as larger equatorial angles lead to lower bandgaps closer to the desired bandgap for solar cells.^15,52^ While it may seem counterintuitive that additional bulkiness leads to less distortion, looking at 3D perovskites provides a logical answer. For MAPbI_3_, the MA^+^ cation is slightly smaller than ideal based on the Goldschmidt tolerance factor which leads to the octahedra tilting in toward each other to a bulkier spacer has the same effect to give less distorted octahedra, though in this case there are no octahedral cages. Here, the spacing cations expand the layers themselves by expanding the half-cage on the surface of the layers. IBA has an even greater effect because it is shorter than IAA, allowing the bulky branched end of the spacer to have more steric influence on the inorganic layer. For *n* = 1 and *n* = 2, this effect may be too extreme, leading to in-plane distortion. Thus, having the right balance of small MA^+^ cations and bulky branched cations leads to Pb–I–Pb angles approaching 180°.

For IAA *n* = 2, the equatorial angles seem to be heavily influenced by its modulated superstructure. As opposed to IBA *n* = 2, IAA *n* = 2 has equatorial angles similar to those of *n* = 3 and 4. Looking closely at the structure, the bond angles can be separated into two categories: those which are along the wave and those which are perpendicular to the wave. The former has an average Pb–I–Pb bond angle of 175.9° and the latter 166.5°, a large difference of nearly 10°. In fact, the angles parallel to the wave have the largest average of any compound reported here while the perpendicular angles are similar to those of BA *n* = 2. Because the ripple wave permeates through the structure, the Pb–I–Pb angles have additional degrees of freedom which allow them to reduce their distortion and approach linearity. This can also be imagined by treating the wave as though it is a distortion which increases the overall distance between the lead atoms. As mentioned earlier, forcing the octahedra away from each other, as is the case for both bulky spacers used here, leads to less distortion as the bond angle is pulled out and straightened. The 8 × 8 supercell here does the same by pulling the lead atoms away from each other, leading to straighter bond angles. This has important applications for band gap tuning, as this reveals another method for decreasing the band gap in optically interesting materials *via* structural modification.

An important effect of the bulkiness of the branched spacer ammonium cations is on the interlayer spacing of the resulting perovskites, defined as the distance between the planes of the terminal iodide atoms of two different layers. Comparing the branched amines to the same-length straight-chain alkylamines (IBA corresponds to PA and IAA to BA) shows that the added bulkiness leads to an increase in interlayer spacing. Looking at the *n* = 4 structures for IAA and BA shows that the straight chain amines pass by each other, but the bulkier IAA amines are sterically prohibited from doing so, pushing the layers apart and leading to an increase in the interlayer spacing from 7.09 Å to 8.82 Å, a difference of 1.73 Å (Fig. 3b and c). This increase is seen in general for all eight structures reported here, though for the IBA system, the effect is not as strong, suggesting the shorter chains allow the layers to get close enough for their attractive forces to overpower the steric hindrance, allowing for some crossover in the IBA system. The IAA *n* = 1 material is a particularly extreme case, as the interlayer spacing is 0.65 Å larger than that for *n* = 2–4. The BA system also shows a larger interlayer spacing for *n* = 1, suggesting that the longer spacers allow the steric hindrance to dominate the attractive forces of thin enough inorganic layers. IAA has the additional length to amplify this effect. Having the bulky cations cause additional repulsion between layers enhances the 2D nature of the Ruddlesden–Popper halide perovskites and makes them promising candidates for studies involving 2D transport as well as exfoliation, as the layers are more weakly bonded and should be easily separated.

### Physical properties

**Fig. 4:**
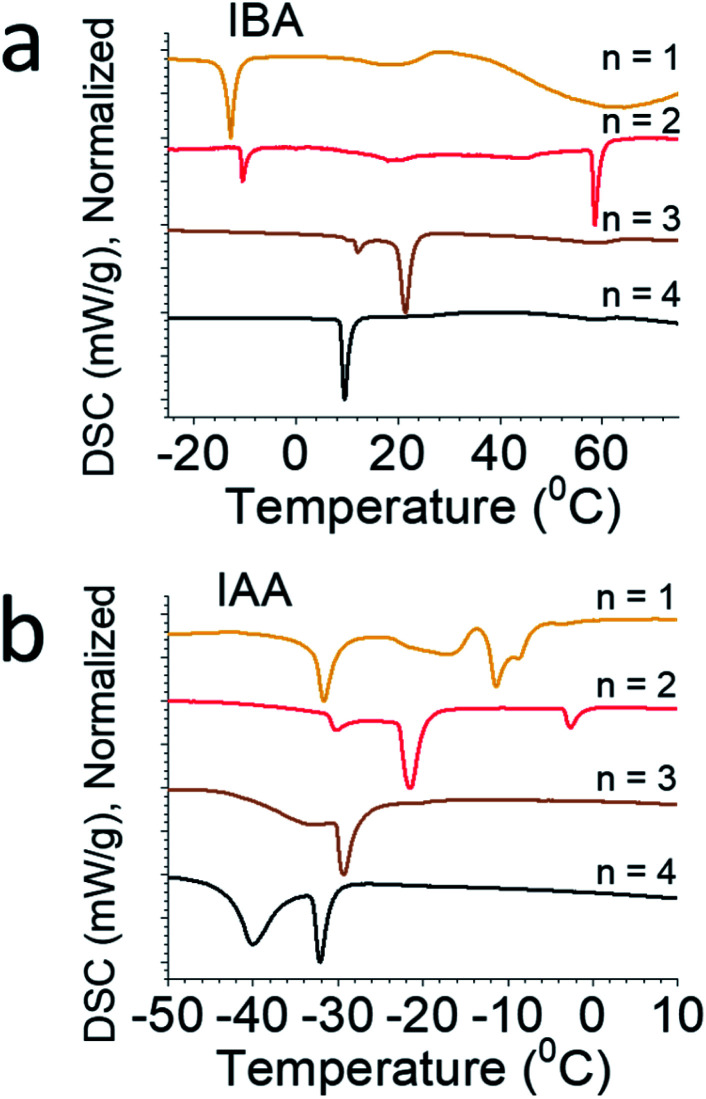
Differential scanning calorimetry (DSC) for (a) IBA and (b) IAA, normalized for clarity.

Differential scanning calorimetry (DSC) was performed on each of the eight compounds (Fig. 4), showing complex phase transitions for several of them. For the straight-chain alkylamines, a single phase transition is seen, caused by a monoclinic to orthorhombic phase transitions as temperature increases. This is seen for the butylammonium, pentylammonium, and hexylammonium spacers. Note that hexylammonium *n* = 1 has a second phase transition, making it an exception to the trends seen for the straight-chain alkylamines.^12,36^ For butylammonium and pentylammonium, the phase transitions are at relatively consistent temperatures when *n* > 1, but for hexylammonium, the transition temperature increases steadily with increasing *n*. In the branched amine system we describe here, multiple phase transitions are seen for nearly every compound. For IBA, *n* = 1 has a single phase transition at −13 °C, but *n* = 2 has two distinct transitions at 10 °C and 59 °C, *n* = 3 has two strong transitions at 12 °C and 23 °C, and *n* = 4 has one strong transition at 10 °C. For IAA, *n* = 1 shows two strong peaks at −31 °C and −11 °C with a few weaker broad peaks as well; IAA *n* = 2 shows three phase transitions at −30 °C, −21 °C, and −3 °C; and IAA *n* = 3 and *n* = 4 have sharp peaks at −29 °C and −32 °C and broad peaks at −33 °C and −39 °C, respectively. Variable-temperature X-ray diffraction (VTXRD) of IAA *n* = 1 shows several peaks split, appear, and disappear, showing the various phase transitions in these materials (Fig. S2†). These systems vary greatly from the straight chain alkylamine systems which highlights the special effect the additional bulkiness of the spacer has on the overall structure stability.

### Optical properties

**Fig. 5:**
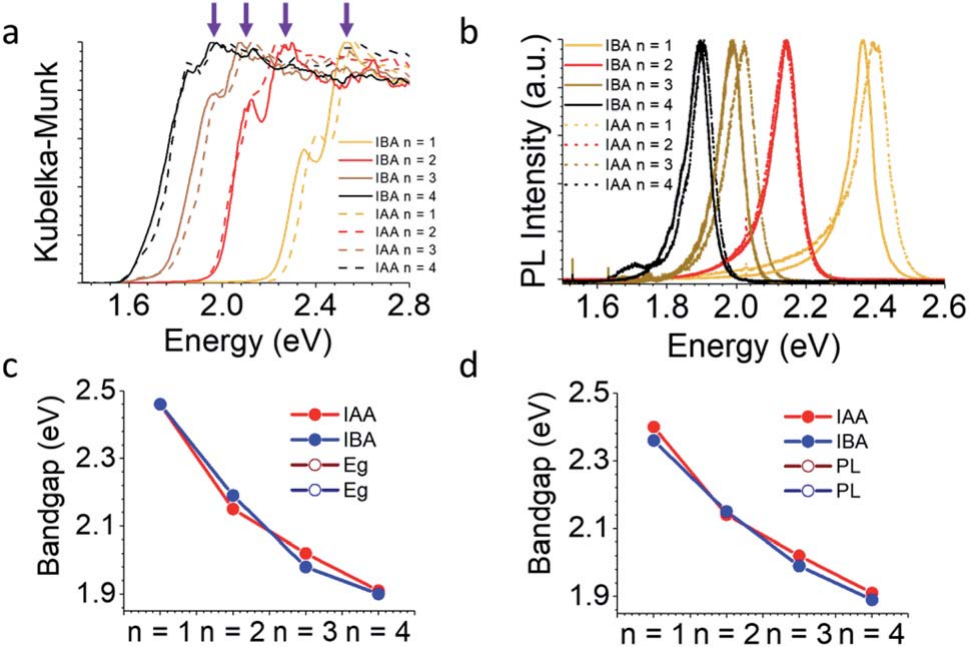
(a) The reflectance spectra for each system transformed *via* the Kubelka–Munk equation. To find the bandgap, the linear portion of the spectra with energy above the bandgap (shown by purple arrows) was extrapolated to the *x*-axis. (b) Photoluminescence for each system. (c and d) A comparison of bandgap and PL, respectively, for the two systems, showing that the IAA is blue-shifted for all *n* values except the modulated structure, *n* = 2.

Phase-pure samples were used to measure the optical properties for both series of compounds. Fig. 5a shows both an absorption edge and a lower energy exciton peak, common for 2D halide perovskites.^34^ The bandgap was estimated by extrapolating the linear region of the absorption edge above the energy of the excitonic peak and determining where it crosses the *x*-axis. The energy of the exciton was determined simply by the position of the exciton peak. It is clear by eye that decreasing *n* leads to a blue shift in the bandgap and exciton energy which is caused by the quantum well structure of these materials. As *n* decreases, the width of the quantum well also decreases, leading to a blue shift, as expected. This trend is also seen in the photoluminescence (PL) data (Fig. 5b). These trends are summarized in Fig. 5c, d and Table S25.† Along with varying *n*, the bandgap can be further tuned using the spacer. The spacer can lead to changes in bandgap for two primary reasons. (1) Uncommonly, if the spacers are short enough, as in the case of DJ and ACI perovskites,^14,15^ the inorganic layers can interact with each other *via* I⋯I contacts, decreasing the degree of quantum confinement and red-shifting the bandgaps. In this case, the interlayer spacing of both IBA and IAA spacers is larger than that of the PA system, which did not obviously show this effect.^43^ (2) The distortions caused by the spacer's interaction with the inorganic layer tunes the Pb–I–Pb angles, which for a given *n* determine the bandgap. As mentioned earlier, the bulkiness of the spacers decreases the distortion for the *n* = 3 and *n* = 4 materials. This should give lower bandgaps for the branched amines in the high *n* structures. Oddly, the IAA system is nearly the same as the BA system, despite its lessened distortions, and the PL is in fact blue-shifted for IAA (Fig. S3†). It is unclear for now whether this could be caused by the increased layer spacing or if some other effect is coming into play here. Compared to IAA, IBA exhibits lower bandgaps and PL for *n* = 1, 3, and 4 presumably because of its decreased distortion. Within the *n* = 2 family the modulated structure for IAA is an exception having lower bandgap than for the IBA *n* = 2 structure (2.15 eV compared to 2.19 eV) for the structure because the modulation leads to larger Pb–I–Pb angles along the wave direction. Similarly, the PL for the *n* = 2 materials shows this inverse trend, though the emissions are closer to the same energy (2.14 eV for IAA and 2.15 eV for IBA).

Along with the bandgap and PL, the exact location of the conduction band minimum (CBM) and valence band maximum (VBM) are of interest when fabricating optoelectronic devices in order to best align the perovskite's energy levels with potential accepting layers. Photoemission yield spectroscopy in air (PYSA) was performed on both systems to find the VBM directly.^53^ The CBM can then be added by summing the VBM and the bandgap.

**Fig. 6:**
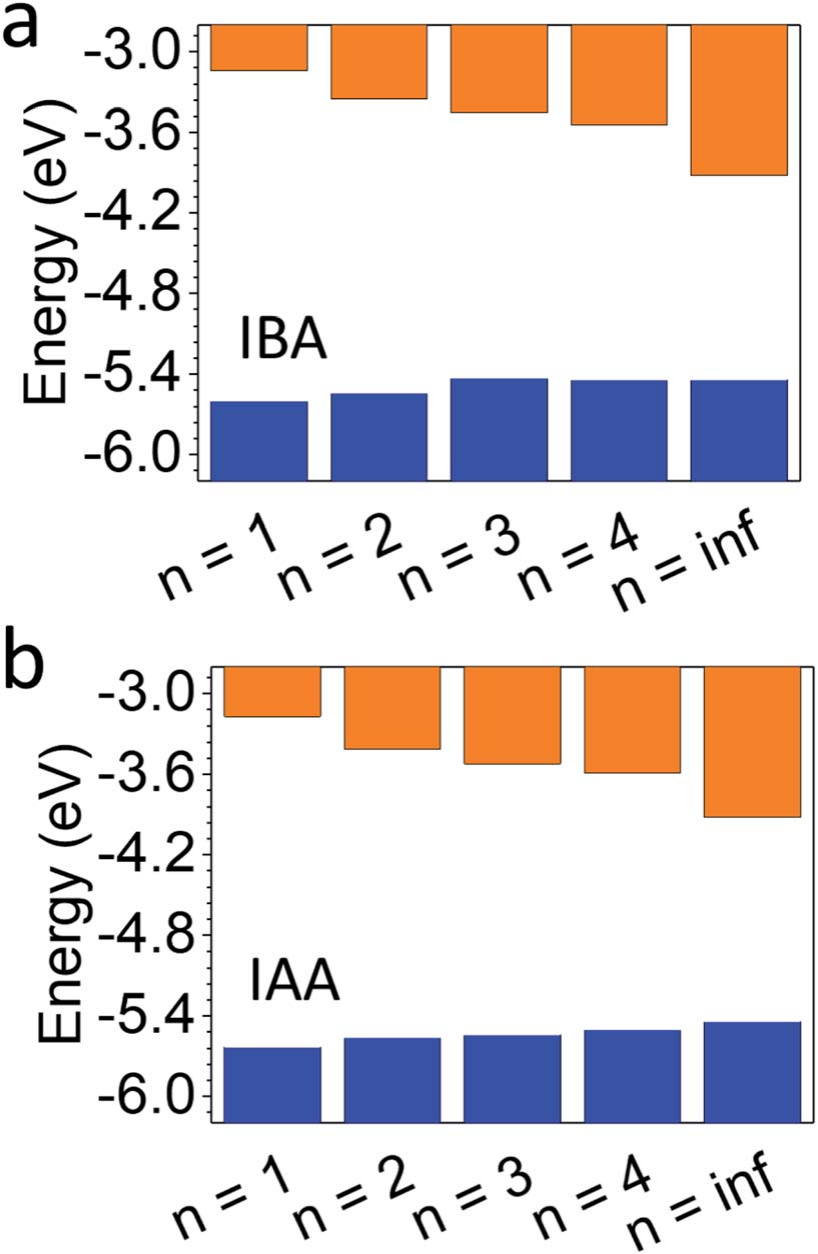
Photoemission yield spectroscopy in air (PYSA) of (a) IBA and (b) IAA, compared to MAPbI_3_ (*n* = inf).

Fig. 6 shows the results with a comparison to MAPbI_3_. For the IAA system, the VBM increases from −5.63 to −5.50 eV as *n* increases from 1 to 4, still noticeably different than the VBM of MAPbI_3_ (−5.44 eV). For the IBA system, however, the VBM increases from −5.60 to −5.44 eV from *n* = 1 to 4, equalling that of MAPbI_3_ by *n* = 4. In fact, the VBM for *n* = 3 is similar to *n* = 4 and MAPbI_3_ in the IBA system. The CBM of both systems shows greater changes with increasing *n* and is still far from MAPbI_3_ for *n* = 4 in each, with IAA *n* = 4 having a CBM of −3.59 eV and IBA *n* = 4 of −3.54 eV, quite different from the CBM for MAPbI_3_ of −3.92 eV. Recently, the straight-chain cations pentylammonium and hexylammonium were studied from members up to *n* = 4.^12^ For this system, the VBM for *n* = 4 in both systems was shown to be near −5.35 eV, higher than that of MAPbI_3_. For the branched amine systems, this anomalous trend is not seen. The CBM of each were closer to that of the branched amines with values near −3.5 eV, suggesting the CBM is less affected by the spacing cation.

### Electronic properties

**Fig. 7:**
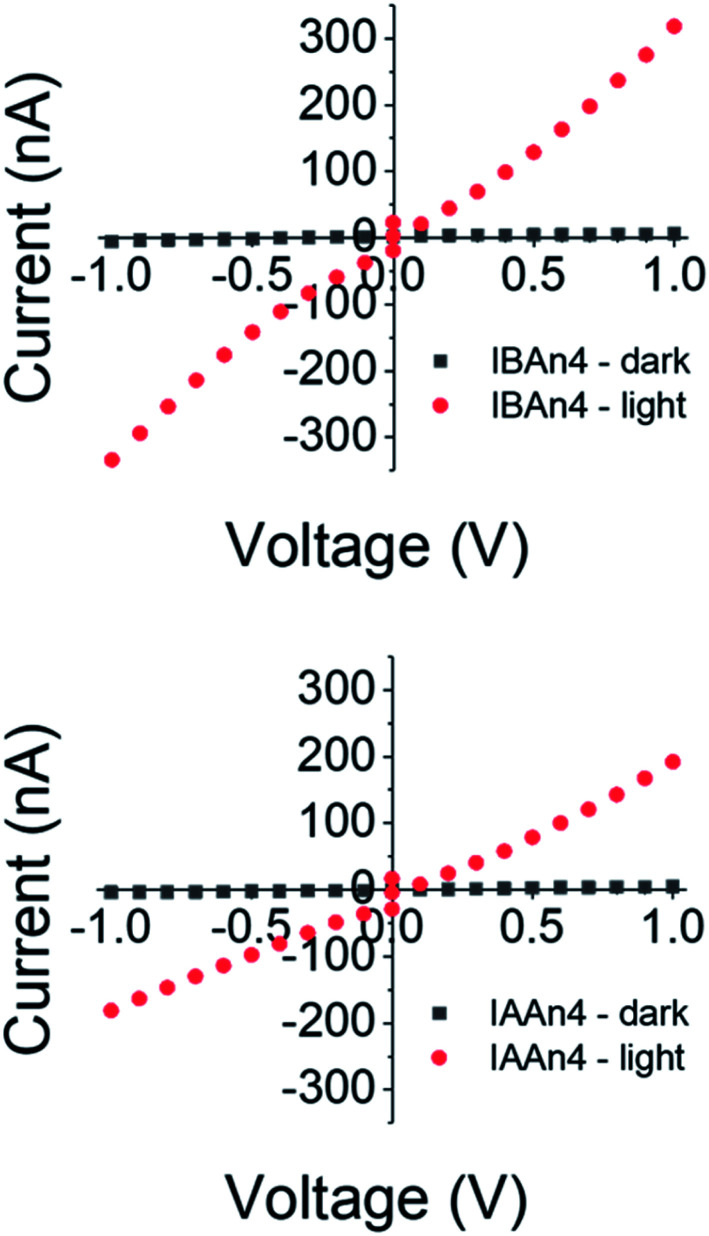
Resistivity measurements of IBA and IAA *n* = 4 crystals parallel to the layers, both in the dark and under white light. Note the strong photovoltaic response for each.

In order to test the utility of these compounds for photovoltaic applications, crystals of *n* = 4 for both IBA and IAA spacers were selected to test for photoresponse using a two-probe setup. These were chosen because previous results have shown higher solar cell efficiencies for *n* > 2.^10^ Copper wires were attached by graphite paste to the edges of the crystals to measure the in-plane response, as this is the plane which carriers are most easily able to flow.^43^ The dark resistivity of both crystals along the plane of the layers was on the order of 10^7^ Ω cm, similar to 3D MAPbI_3_(ref. 54) (Fig. 7 and S5†). Furthermore, upon white light illumination, the resistivity decreased to approximately 10^5^ Ω cm, showing a clear photoresponse, warranting these materials to be tested in solar cell devices. Note that the IAA and IBA compounds showed very similar conductivity and responses, indicating that any differences in the device performance is not based on their ability to conduct photoexcited carriers.

### Film characterization and devices

With the *n* = 4 crystals showing good photoresponse along with the appropriate bandgap, films were made to further probe their use in devices. A simple one-step spincoating method was used to deposit the films, which was followed by annealing 10 min at 100 °C. To some films 2.5 wt% MACl was added, which has been shown to improve the crystallinity of the films and thus the efficiency of the resulting devices.^55^ Films were deposited on PEDOT:PSS to simulate the conditions when fabricating a photovoltaic device. The films for each spacer, with and without MACl, were analysed to see the effects of the spacer and the additive. Each of the films is around 230 nm thick as seen by cross-section scanning electron microscopy (SEM, Fig. S6†). The morphology was analysed with atomic force microscopy (AFM, Fig. S7†). Using IAA appeared to give smoother films than when IBA was used, with or without MACl having been added. A change of morphology can also be seen upon addition of MACl. The grains become more distinct, especially for IAA, though IBA shows a significant degree of roughness.

**Fig. 8:**
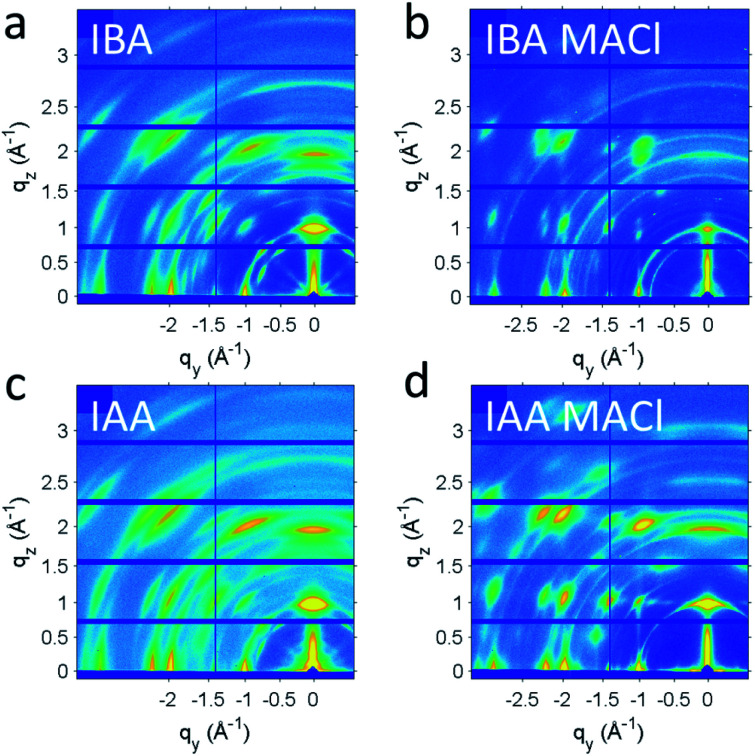
(a and c) GIWAXS patterns for IAA and IBA *n* = 4 films using one-step spincoating. (b and d) The films with an MACl additive show less broadening along *χ*, indicative of more preferred orientation.

To better understand the crystallinity and orientation of the grains, grazing-incident wide-angle X-ray scattering (GIWAXS) measurements were taken (Fig. 8). To help facilitate charge transport in solar cell devices, orientation of the layer perpendicular to the substrate is desired.^9^ Without addition of MACl, both IAA *n* = 4 and IBA *n* = 4 films show rings with very broad spots, indicating a low degree of preferred orientation. After addition of MACl, distinct spots appear for both, though weak rings remain in the background, indicative that not all grains are oriented. To better quantify the orientation and crystallinity, linecuts along *q* and *χ* were taken, in which *χ* is the angle in polar coordinates of the GIWAXS pattern. For the crystallinity, the full-width half-maximum (FWHM) of the linecut along *q* of the (0 8 1) peak was used. The (0 8 1) peak was chosen because it is not hindered by other peaks or geometric limitations. The MACl appears to have given both samples an increased degree of crystallinity, as can be seen by the decrease in FWHM along *q* for each peak. Specifically, IBA sees a decrease in FWHM from 0.084 Å without MACl to 0.067 Å with MACl, and IAA sees a decrease from 0.082 Å to 0.061 Å, indicating that IBA and IAA do not differ much in crystallinity. To quantify the preferred orientation, an arc – cut along *χ*, also for the (0 8 1) peak, was fitted to a Gaussian curve to show the distribution of different orientations with the fitted *σ* value representing the standard deviation of the orientations from perpendicular orientation. Both films exhibited improvements in orientation with addition of MACl, IBA going from a *σ* value of 3.73° to 1.64° and IAA from 4.11° to 3.44°. These values indicate that these are promising films to be incorporated into solar cell devices.

**Fig. 9:**
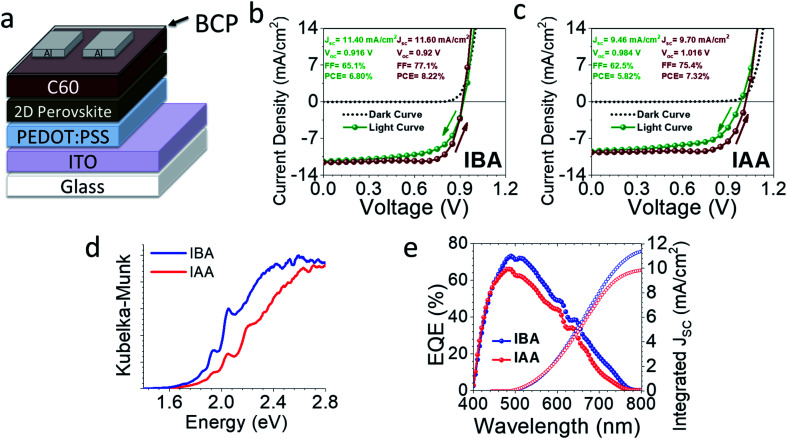
(a) The architecture used for these devices. “2D Perovskite” refers to either IAA or IBA *n* = 4 with 2.5 wt% MACl added. (b and c) Representative device curves for each material, showing scans in both directions. (d) The reflectance data of each film transformed by the Kubelk–Munk equation, showing the appearance of various n peaks. (e) The EQE spectrum of representative devices for each material.

Preliminary devices were made using an inverted architecture ITO/PEDOT:PSS/perovskite/C60/BCP/Al (Fig. 9). All devices were made using the MACl additive, as discussed above. The champion device curves are seen in Fig. 9b and c, and a full summary is seen in Fig. S9 and Table S26.† IBA gave a champion efficiency of 8.22% with average values of 7.45 ± 0.53% while IAA gave slightly a lower efficiency of 7.32% with average values of 6.13 ± 0.73%. IBA appears to be limited by its *V*_OC_, likely caused by the poor film morphology based on AFM. That being said, it has a decent current density (*J*_SC_) above 11 mA cm^−2^ on average compared to that of IAA, which average closer to 9 mA cm^−2^. This is likely due to the perovskite layers mostly being perpendicularly oriented, allowing for good carrier extraction. The IBA system gives small interlayer spacings compared to BA, which causes a higher percentage of the volume to be taken up by the absorbing inorganic layers, leading to higher absorption. This is clear from the external quantum efficiency (EQE) curves and bandgaps in Fig. 9. The shape of each is similar, indicating the same wavelengths of light are being absorbed but the IBA-based device absorbs more light, likely a result of the smaller spacer size. Based on these preliminary results, both cations have potential use in future solar cell devices, especially once further optimization is done.

## Conclusions

The bulky branched spacers IBA and IAA stabilize eight 2D RP perovskite structures with the general formula (RA)_2_(MA)_*n*−1_Pb_*n*_I_3*n*+1_ (RA = IBA, IAA; *n* = 1–4). The IAA *n* = 2 member exhibits the first observed modulated 2D halide perovskite structure. In general, these two homologous series show less distortion than the straight-chained alkyl amines of the same length, which results from the additional bulkiness of the spacers. Even though MA in the cages is too small to stabilize an ideal perovskite structure, the bulky spacers compensate for this, as FA compensates for MA in 3D materials. The modulated structure is a new feature in halide perovskite chemistry and adds another degree of tunability by stretching the octahedra along the direction of the wave, pulling the bond angles toward linearity. All these factors play a role in reducing the bandgap of the materials, especially IBA which is the bulkier of the two cations. This understanding of the interaction between organic and inorganic layers and structure–property relationships gives key insight to how the structure can be optically tuned for improve device performance. The ability of these materials to be used in solar cell devices was confirmed by a photoresponse occurring along the layers of crystals of *n* = 4 for both spacers. Films with 2.5 wt% MACl added were fabricated and analyzed with GIWAXS, which showed high orientation with the layers perpendicular to the substrate, ideal for devices. As proof-of-concept, solar cell devices were designed and tested, showing efficiencies of 7.45 ± 0.53% for IBA and 6.13 ± 0.73% for IAA. Further tuning the film fabrication and improved device architectures will improve these efficiencies, as the excellent preferred orientation, optical properties, and photoresponse of each system gives high potential for efficient solar cell devices. The use of these materials in 2D/3D devices can also be considered based on this analysis.^56,57^ Particularly, the small interlayer spacing in the IBA system is promising for better solar cell efficiencies. For some time, researchers have expected that the halide perovskite structure is flexible and able to accommodate strain, this is the first structurally documented example where we can see that this is indeed the case as demonstrated by the curvature of the inorganic slabs. This could help to carry out more informed modeling studies on the mechanoelastic/electronic property relationships in these materials. Further understanding of how other modulated structures could be found could open up more possibilities in fine tuning the properties of 2D halide perovskites.

## Author contributions

J. M. H. and M. G. K. conceived the idea and designed the study. J. M. H. performed the synthesis, physical characterization, optical measurements, resistivity, GIWAXS, and film characterization. C. D. M. refined the structure for the modulated IAA *n* = 2 compound while J. M. H. refined the other structures. S. S. made and characterized the solar cells. R. M. performed *in situ* diffraction experiments. The manuscript was written by J. M. H. and M. G. K. All authors discussed the results and commented on the manuscript.

## Conflicts of interest

There are no conflicts to declare.

## Supplementary Material

SC-011-D0SC04144K-s001

SC-011-D0SC04144K-s002
