# Supplementary material for: Long periodic ripple in a 2D hybrid halide perovskite structure using branched organic spacers
Source: Chem Sci. 2020 Oct 6;11(44):12139–48. doi: 10.1039/d0sc04144k (PMC8162985; doi:10.1039/d0sc04144k)
Supplement: SC-011-D0SC04144K-s001 [file SC-011-D0SC04144K-s001.pdf]

Electronic Supplementary Material (ESI) for Chemical Science.
This journal is © The Royal Society of Chemistry 2020

Supporting Information

Long Periodic Ripple in a 2D Hybrid Halide Perovskite Structure Using Branched Organic Spacers

Justin M. Hoffman,[1] Christos D. Malliakas,[1] Siraj Sidhik,[2] Ido Hadar,[1] Rebecca McClain,[1] Aditya D. Mohite,[2] and Mercouri G. Kanatzidis[1]*

E-mail: m-kanatzidis@northwestern.edu

[1] Department of Chemistry, Northwestern University, Evanston, IL 60208, United States
[2] Department of Chemical and Biomolecular Engineering, Rice University, Houston, Texas 77005, United States

Experimental

Synthesis.

$(IBA)_2PbI_4$. PbO (2.232 g, 10 mmol) was dissolved in a mixture of 30.0 mL aqueous HI and 1.7 mL aqueous H_3PO_2 by heating at 190 ºC under constant magnetic stirring for 5 min. In a separate vial, 5 mL aqueous HI was cooled in an ice bath. Then, IBA (1.9876 mL, 20 mmol) was added slowly and allowed 5 min to fully neutralize. To the hot bright yellow solution was added the neutralized IBA. The resulting yellow solution was stirred for 5 min. Then, the stirring was ceased and the solution was left on the hotplate, which was turned off and slowly returned to room temperature, giving bright orange plates. After five hours, crystallization was considered complete, and the plates were dried through vacuum filtration for 30 min before being dried under vacuum overnight. Yield 8.24g (95.5% based on mole Pb).

$(IBA)_2(MA)Pb_2I_7$. PbO (2.232 g, 10 mmol) and MACl (338.0 mg, 5 mmol) were dissolved in a mixture of 13.0 mL aqueous HI and 1.7 mL aqueous H_3PO_2 by heating at 190 ºC under constant magnetic stirring for 5 min. In a separate vial, 2 mL aqueous HI was cooled in an ice bath. Then, IBA (497.1 µL, 5 mmol) was added slowly and allowed 5 min to fully neutralize. To the hot bright yellow solution was added the neutralized IBA, giving an orange precipitate which redissolved quickly. The resulting yellow solution was stirred for 5 min. Then, the stirring was ceased and the solution was left on the hotplate, which was turned off and slowly returned to room temperature, giving red plates. After five hours, crystallization was considered complete, and the plates were dried through vacuum filtration for 30 min before being dried under vacuum overnight. Yield 3.0 g (41% based on mole Pb).

$(IBA)_2(MA)_2Pb_3I_{10}$. PbO (2.232 g, 10 mmol) and MACl (450.0 mg, 6.67 mmol) were dissolved in a mixture of 12.0 mL aqueous HI and 1.7 mL aqueous H_3PO_2 by heating at 190 ºC under

constant magnetic stirring for 5 min. In a separate vial, 2 mL aqueous HI was cooled in an ice bath. Then, IBA (331.2 µL, 3.33 mmol) was added slowly and allowed 5 min to fully neutralize. To the hot bright yellow solution was added the neutralized IBA, giving a black precipitate which redissolved quickly. The resulting yellow solution was stirred for 5 min. Then, the stirring was ceased and the solution was left on the hotplate, which was turned off and slowly returned to room temperature, giving dark red plates. After five hours, crystallization was considered complete, and the plates were dried through vacuum filtration for 30 min before being dried under vacuum overnight. Yield 2.4 g (34% based on mole Pb).

$(IBA)_2(MA)_3Pb_4I_{13}$. PbO (2.232 g, 10 mmol) and MACl (507.4 mg, 7.5 mmol) were dissolved in a mixture of 12.0 mL aqueous HI and 1.7 mL aqueous H_3PO_2 by heating at 190 ºC under constant magnetic stirring for 5 min. In a separate vial, 2 mL aqueous HI was cooled in an ice bath. Then, IBA (248.4 µL, 2.5 mmol) was added slowly and allowed 5 min to fully neutralize. To the hot bright yellow solution was added the neutralized IBA, giving a black precipitate which redissolved quickly. The resulting yellow solution was stirred for 5 min. Then, the stirring was ceased and the solution was left on the hotplate, which was turned off and slowly returned to room temperature, giving black plates. After five hours, crystallization was considered complete, and the plates were dried through vacuum filtration for 30 min before being dried under vacuum overnight. Yield 3.0 g (45% based on mole Pb).

$(IAA)_2PbI_4$. PbO (2.232 g, 10 mmol) was dissolved in a mixture of 30.0 mL aqueous HI and 1.7 mL aqueous H_3PO_2 by heating at 190 ºC under constant magnetic stirring for 5 min. In a separate vial, 5 mL aqueous HI was cooled in an ice bath. Then, IAA (2.3212 mL, 20 mmol) was added slowly and allowed 5 min to fully neutralize, leaving a white precipitate at the top of the solution, which was then heated briefly at 100 ºC to allow the precipitate to dissolve. To the hot

bright yellow solution was added the neutralized IAA. The resulting yellow solution was stirred for 5 min. Then, the stirring was ceased and the solution was left on the hotplate, which was turned off and slowly returned to room temperature, giving light orange plates. After five hours, crystallization was considered complete, and the plates were dried through vacuum filtration for 30 min before being dried under vacuum overnight. Yield 6.88 g (77% based on mole Pb).

$(IAA)_2(MA)Pb_2I_7$. PbO (2.232 g, 10 mmol) and MACl (338.0 mg, 5 mmol) were dissolved in a mixture of 13.0 mL aqueous HI and 1.7 mL aqueous H_3PO_2 by heating at 190 ºC under constant magnetic stirring for 5 min. In a separate vial, 2 mL aqueous HI was cooled in an ice bath. Then, IAA (580.4 μL, 5 mmol) was added slowly and allowed 5 min to fully neutralize, leaving a white precipitate at the top of the solution, which was then heated briefly at 100 ºC to allow the precipitate to dissolve. To the hot bright yellow solution was added the neutralized IAA. The resulting yellow solution was stirred for 5 min. Then, the stirring was ceased and the solution was left on the hotplate, which was turned off and slowly returned to room temperature, giving bright red plates. After three hours, crystallization was considered complete, and the plates were dried through vacuum filtration for 30 min before being dried under vacuum overnight. Yield 2.63 g (35% based on mole Pb).

$(IAA)_2(MA)_2Pb_3I_{10}$. PbO (2.232 g, 10 mmol) and MACl (450.0 mg, 6.67 mmol) were dissolved in a mixture of 12.0 mL aqueous HI and 1.7 mL aqueous H_3PO_2 by heating at 190 ºC under constant magnetic stirring for 5 min. In a separate vial, 2 mL aqueous HI was cooled in an ice bath. Then, IAA (388.0 μL, 3.33 mmol) was added slowly and allowed 5 min to fully neutralize, leaving a white precipitate at the top of the solution, which was then heated briefly at 100 ºC to allow the precipitate to dissolve. To the hot bright yellow solution was added the neutralized IAA. The resulting yellow solution was stirred for 5 min. Then, the stirring was ceased and the

solution was left on the hotplate, which was turned off and slowly returned to room temperature, giving dark red plates. After five hours, crystallization was considered complete, and the plates were dried through vacuum filtration for 30 min before being dried under vacuum overnight. Yield 2.76 g (39% based on mole Pb).

$(IAA)_2(MA)_3Pb_4I_{13}$. PbO (2.232 g, 10 mmol) and MACl (507.4 mg, 7.5 mmol) were dissolved in a mixture of 12.0 mL aqueous HI and 1.7 mL aqueous H_3PO_2 by heating at 190 ºC under constant magnetic stirring for 5 min. In a separate vial, 2 mL aqueous HI was cooled in an ice bath. Then, IAA (290.4 μL, 2.5 mmol) was added slowly and allowed 5 min to fully neutralize, leaving a white precipitate at the top of the solution, which was then heated briefly at 100 ºC to allow the precipitate to dissolve. To the hot bright yellow solution was added the neutralized IAA. The resulting yellow solution was stirred for 5 min. Then, the stirring was ceased, and the solution was left on the hotplate, which was turned off and slowly returned to room temperature, giving black plates. After five hours, crystallization was considered complete, and the plates were dried through vacuum filtration for 30 min before being dried under vacuum overnight. Yield 2.70g (39% based on mol Pb).

Materials Characterization

Single-crystal X-ray diffraction experiments were performed using one of the following diffractometers: a STOE IPDS II, IPDS 2T, or Rigaku Mo-Synergy diffractometer with Mo Kα radiation (λ = 0.71073 Å) and operating at 50 kV and 40 mA. Integration and numerical absorption corrections were performed using the X-AREA, X-RED, and X-SHAPE programs. The structures were solved by charge flipping and refined by full-matrix least-squares on F2

using the Jana2006 package.[1] The PLATON[2] software was used to identify the twinning domains and validate the space groups of the compounds.

A STOE STADI MP high-resolution diffractometer with an Oxford Cryostream 700 Plus attachment was used to collect temperature-dependent powder X-ray diffraction data. The diffractometer was equipped with a one-dimensional silicon strip detector (MYTHEN2 1K from DECTRIS) and an asymmetric curved Germanium monochromator. The sample, IAA, was packed into a 0.8 mm diameter Kapton tube and sealed with epoxy. Diffraction data was collected at 20°C, every 2°C from 0°C to -35°C, -40°C, and -45°C for both heating and cooling. The sample was heated and cooled at a rate of 2°C/min and spun throughout the collection. Diffraction data was collected using copper Kα1 radiation (1.54060 Å) operated at 40 kV and 40 mA. Prior to measurement, the instrument was calibrated against a NIST Silicon standard (640d).

Differential scanning calorimetry (DSC) measurements were performed on a Netzsch's Simultaneous Thermal Analysis (STA) system. About 20-30 mg of sample were placed in sealed aluminum pans. Measurements were performed under He gas at a scan rate of 10 °C/min.

A Shimadzu UV-3600 PC double-beam, double-monochromator spectrophotometer was used to acquire room-temperature optical diffuse reflectance spectra of the powdered samples in the range of 200−2500 nm. $BaSO_4$ was used as a non-absorbing reflectance reference, and reflectance data were converted to absorbance data via Kubelka−Munk transformation.[3] Both bandgaps and exciton energies were extracted from the data. The exciton energies were estimated based on the position of the exciton peak residing below the bandgap. The linear portion of the curve above the exciton was used to extract the bandgap based on its intersection with the x-axis. Low-energy impurity peaks, appearing as tails in the spectra, were ignored.

Steady-state PL spectra were collected using HORIBA LabRAM HR Evolution confocal Raman microscope (600 g/mm diffraction grating) equipped with a Synapse charge-coupled device camera. A diode continuous wave laser (473 nm, 25 mW) filtered at 0.01-0.1% power was used to excite all samples at 50× magnification.

The VBM of the powders was measured by Photoemission Yield Spectroscopy in Air (PYSA) measurements, AC-2 Riken-Keiki. Briefly the samples were scanned by monochromatic UV light (4.2-6.2 eV), under ambient conditions and the number of generated photoelectrons were measured at each energy. Photoelectrons are only generated when the photon energy is higher than the VBM, hence the VBM is determined by finding the onset of the PYSA spectrum.[4]

Current-voltage (I-V) curves were measured at room temperature in air using a Keithley 6517b picoammeter/voltage supply under -10 to 10 V bias via a two-probe method. Devices were made using single crystals of the compounds. Electrical contacts were applied through 100 μm copper wires adhered to the crystal specimens through colloidal graphite paste. Measurements were made on single crystals with contacts connecting on the edges of the crystals. The devices were put inside a guarded dark box for dark measurements. Additionally, they were measured under a white light source with a power setting of 7 W (Taotronics TT-DL11). Resistivity was calculated using the equation $\rho = R * \frac{A}{l}$ where ρ is resistivity, R is resistance calculated by the slope of the I-V curve collected above, and A and l are the cross-sectional area of the crystal and the length from one contact to the other. It was assumed that the contact resistance was similar for each crystal.

Thin Film Characterization

Thin films were prepared in a nitrogen atmosphere on glass substrates coated with PEDOT:PSS. Oven-dried crystals were dissolved in DMF (0.60 M) with 2.5 %wt MACl added. Solutions were stirred at 70 ºC for six hours and spin coated 30 s at 4000 rpm then annealed 10 minutes at 100 ºC. The thickness was measured using a high-resolution field emission scanning electron microscope (Hitachi SU8030). XRD measurements were carried out on a Rigaku MiniFlex600 X-ray diffractometer (Cu Kα radiation, λ = 1.5406 Å) operating at 40 kV and 15 mA. GIWAXS measurements were performed at Beamline 8-ID-E of the Advanced Photon Source at Argonne National Laboratory. Samples prepared on glass substrates were exposed to an X-ray beam (λ = 1.14 Å) at an incident angle of 0.15° for 5 s, and the scattered light was collected by a Pilatus 1 M pixel array detector at 204 mm from the sample. The GIXSGUI program was used to plot images of the patterns and analyze the data.[5]

Solar Cell Device Fabrication

The ITO-coated glass substrates (TEC7, 2.2 mm, Hartford Glass Co. Inc.) were cleaned by sequential sonication in aqueous detergent, deionized water, acetone, and isopropyl alcohol for 10 min each, followed by a 3 min oxygen plasma treatment. An aqueous suspension (1.75% solid content) of PEDOT:PSS was deposited on the pre-cleaned ITO substrates by spin-coating at 6000 rpm for 30 s and annealed at 150 °C for 30 min in air. The substrates were then transferred to an argon-filled glovebox to complete the rest of the device fabrication. Oven-dried crystals of $(IAA)_2(MA)_3Pb_4I_{13}$ and $(IBA)_2(MA)_3Pb_4I_{13}$ were dissolved in DMF at a concentration of 0.60 M Pb^{2+} with 2.5 %wt MACl added. Solutions were stirred at 70 ºC for six hours and spin coated 30 s at 4000 rpm then annealed 10 minutes at 100 ºC. The C_{60} (80 nm) layer was deposited on the perovskite film using thermal deposition. Finally, 100 nm of Ag was thermally

evaporated through a shadow mask at a pressure of ~1×10^{-6} Torr. The active area of the device was 0.09 cm^2.

Solar Cell Device Characterization

The current density versus voltage (J−V) characteristics were collected in air using a Keithley 2400 source meter under simulated AM 1.5G irradiation (100 mW/cm^2) generated by a standard solar simulator (Abet Technologies). The light intensity was calibrated by using an NREL-certified monocrystalline Si reference cell to reduce the spectral mismatch. The external quantum efficiencies (EQE) were collected by illuminating the device under monochromatic light using a tungsten source (chopped at 150 Hz) while collecting the photocurrent by lock-in amplifier in AC mode. The light source spectrum response was corrected by calibrated silicon diode (FDS1010, Thorlab).

General information and refinement in superspace

The distortion (positional or temperature parameter) of a given atomic parameter x_4 in the subcell can be expressed by a periodic modulation function $p(x_4)$ in a form of a Fourier expansion:

$$p(k + x_4) = \sum_{n=1}^{m} A_{sn} \sin[2\pi\overline{q}_n (k + x_4)] + \sum_{n=1}^{m} A_{cn} \cos[2\pi\overline{q}_n (k + x_4)]$$

where A_{sn} is the sinusoidal coefficient of the given Fourier term, A_{cn} the cosine coefficient, *n* the number of modulation waves used for the refinement and *k* the lattice translation. $\overline{q}_n = \sum_{i=1}^{d} \alpha_{ni} q_i$ where α_{ni} integer numbers for the linear combination of the incommensurate modulation vectors q_i.

The length determination and refinement of the q-vectors was performed with the Peaklist 1.06 software part of the X-Area 1.39 suite (X-AREA, IPDS Software, STOE & Cie GmbH, Darmstadt, 2006) using a least square refinement algorithm.

Satellite reflections of first order were observed and used for the refinement. A single modulation wave for positional and temperature parameters was used. Only the symmetry allowed Fourier terms were refined. In more details, the sinusoidal coefficients in all the directions for positional (x, y and z) and thermal parameters (U_{11}, U_{22}, U_{33}, U_{12}, U_{13} and U_{23}) were freely refined and the corresponding cosine coefficients values were induced by symmetry.

Position of the organic molecules in the modulated structure was not determined/refined due to disorder of the organic molecules.

Table S1. Atomic coordinates (x10^4) and equivalent isotropic displacement parameters (Å^2 x 10^3) for IBA n = 1 at 293 K with estimated standard deviations in parentheses.

Label	x	y	z	Occupancy	U_{eq}*
Pb(1)	5000	5000	0	1	37(1)
I(1)	4899(1)	1998(1)	1933(1)	1	52(1)
I(2)	7418(1)	4630(1)	1352(1)	1	61(1)
N(1)	7003(7)	751(11)	256(11)	1	106(2)
C(1a)	7463(6)	-440(10)	1462(12)	1	106(2)
C(1b)	8562(6)	-85(7)	2291(9)	1	106(2)
C(1c)	9036(8)	-1315(13)	3437(12)	1	106(2)
C(1d)	9110(8)	64(14)	1070(13)	1	106(2)
H(1c1a)	7105.63	-485.93	2238.31	1	127.5
H(2c1a)	7411.83	-1381.75	927.44	1	127.5
H(1c1b)	8612.1	832.38	2869.94	1	127.5
H(1c1c)	8565.14	-1644.71	3969.1	1	127.5
H(2c1c)	9202.51	-2129.91	2857.72	1	127.5
H(3c1c)	9644.95	-955.6	4217.54	1	127.5
H(1c1d)	9587.01	870.64	1363.88	1	127.5
H(2c1d)	9466.41	-843.25	1025.74	1	127.5
H(3c1d)	8625.79	260.08	37.19	1	127.5
H(1n1)	6502.03	377.6	-514.68	1	127.5
H(2n1)	6776.62	1467.28	718.1	1	127.5
H(3n1)	7462.77	1097.51	-135.96	1	127.5

*U_{eq} is defined as one third of the trace of the orthogonalized U_{ij} tensor.

Table S2. Anisotropic displacement parameters ($Å^2$ x 10^3) for IBA n = 1 at 293 K with estimated standard deviations in parentheses.

Label	U_{11}	U_{22}	U_{33}	U_{12}	U_{13}	U_{23}
Pb(1)	50(1)	31(1)	33(1)	0(1)	16(1)	0(1)
I(1)	72(1)	44(1)	41(1)	-10(1)	17(1)	13(1)
I(2)	51(1)	66(1)	66(1)	-7(1)	19(1)	-8(1)

The anisotropic displacement factor exponent takes the form: $-2\pi^2[h^2a^{*2}U_{11} + ... + 2hka^*b^*U_{12}]$.

Table S3. Selected bonds and angles for IBA n = 1 at 293 K with estimated standard deviations in parentheses.

Label	Distances (Å)	Label	Angles (°)
Pb(1)-I(1)	3.2139(6)	Pb(1)-I(1)-Pb(1)#6	156.477(18)
Pb(1)-I(1)#1	3.2037(6)		
Pb(1)-I(1)#2	3.2139(6)		
Pb(1)-I(1)#3	3.2037(6)		
Pb(1)-I(2)	3.1975(7)		
Pb(1)-I(2)#2	3.1975(7)		

Table S4. Atomic coordinates (x10^4) and equivalent isotropic displacement parameters (Å^2 x 10^3) for IBA n = 2 at 293 K with estimated standard deviations in parentheses.

Label	x	y	z	Occupancy	U_{eq}^*
Pb(1)	4164(1)	2486(1)	2503(1)	1	41(1)
I(1)	5000	2418(3)	2500	1	71(1)
I(2)	792(1)	435(2)	437(2)	1	67(1)
I(3)	9160(1)	4553(2)	449(2)	1	74(1)
I(4)	6634(1)	2853(2)	2388(2)	1	81(1)
C(2a)	6645(6)	8010(30)	2230(40)	1	163(7)
N(2)	6480(7)	6870(30)	3250(40)	1	163(7)
C(2c)	7193(7)	8840(40)	1170(40)	1	163(7)
C(2b)	7016(6)	7610(20)	2010(30)	1	163(7)
C(1a)	10074(13)	2890(40)	1720(20)	1	290(20)
C(2d)	7186(7)	7390(40)	3510(30)	1	163(7)
H(1c2a)	6629.65	8979.12	2686.29	1	195.1
H(2c2a)	6529.69	8013.83	1273.93	1	195.1
H(1c2c)	7044.45	9700.99	1119.71	1	195.1
H(2c2c)	7401.29	9107.17	1682.6	1	195.1
H(3c2c)	7245.51	8516.22	161.2	1	195.1
H(1c2b)	7031.5	6698.61	1433.81	1	195.1
H(1c2d)	7349.35	6593.82	3449.01	1	195.1
H(2c2d)	7300.94	8298.84	3801.57	1	195.1
H(3c2d)	7014.84	7150.91	4255.56	1	195.1

H(1n2)	6360.2	7330.93	3935.62	1	195.1
H(2n2)	6346.09	6298.33	2717.31	1	195.1
H(3n2)	6638.37	6334.82	3680	1	195.1
N(1a)	10074(13)	2890(40)	1720(20)	0.5	290(20)
H(1n1)	10104.95	1977.1	1420.5	0.5	344
H(2n1)	9932.49	3342.2	1104.45	0.5	344
H(3n1)	10269.53	3363.65	1722.1	0.5	344

*U_{eq} is defined as one third of the trace of the orthogonalized U_{ij} tensor.

Table S5. Anisotropic displacement parameters ($Å^2 \times 10^3$) for IBA n = 2 at 293 K with estimated standard deviations in parentheses.

Label	U_{11}	U_{22}	U_{33}	U_{12}	U_{13}	U_{23}
Pb(1)	53(1)	31(1)	40(1)	0(1)	0(1)	0(1)
I(1)	46(2)	82(2)	85(2)	0	-2(1)	0
I(2)	90(1)	50(1)	62(1)	5(1)	1(1)	27(1)
I(3)	105(2)	52(1)	64(1)	-15(1)	13(1)	-30(1)
I(4)	51(1)	85(2)	106(2)	5(1)	-3(1)	-12(1)

The anisotropic displacement factor exponent takes the form: $-2\pi^2[h^2a^{*2}U_{11} + ... + 2hka^*b^*U_{12}]$.

Table S6. Selected bonds and angles for IBA n = 2 at 293 K with estimated standard deviations in parentheses.

Label	Distances (Å)	Label	Angles (°)
Pb(1)-I(1)	3.2536(7)	Pb(1)-I(1)-Pb(1)#5	177.85(9)
Pb(1)-I(2)#1	3.2097(16)	Pb(1)#11-I(2)-Pb(1)#2	159.28(6)
Pb(1)-I(2)#2	3.2034(16)	Pb(1)#12-I(3)-Pb(1)#13	159.68(6)
Pb(1)-I(3)#3	3.1916(16)		
Pb(1)-I(3)#4	3.1822(17)		
Pb(1)-I(4)#5	3.1278(17)		

Table S7. Atomic coordinates (x10^4) and equivalent isotropic displacement parameters (Å^2 x 10^3) for IBA n = 3 at 293 K with estimated standard deviations in parentheses.

Label	x	y	z	Occupancy	U_{eq}*
Pb(1)	2(2)	2524(4)	1247(1)	1	52(1)
Pb(2)	0	2526(7)	2500	1	48(1)
I(1)	2592(8)	57(11)	2501(3)	1	141(3)
I(2)	2584(5)	25(7)	1262(2)	1	137(5)
I(3)	2408(6)	5031(8)	1260(2)	1	144(6)
I(4)	-3(7)	2629(14)	1887(1)	1	178(3)
I(5)	-25(10)	2640(20)	656(2)	1	277(6)
N(1)	4720(80)	2020(80)	767(12)	1	270(20)
C(1a)	5980(70)	1500(80)	596(16)	1	270(20)
N(2)	1080(60)	6580(60)	1829(12)	1	160(20)
C(1b)	5660(60)	1920(70)	317(14)	1	270(20)
C(1c)	6450(80)	3350(90)	252(17)	1	270(20)
C(1d)	4010(60)	2110(120)	280(15)	1	270(20)
H(1c1a)	6892.65	1970.87	651.56	1	329.7
H(2c1a)	6078.75	436.44	609.79	1	329.7
H(1c2)	-25.54	8557.89	1848.54	1	189.3
H(2c2)	-1046.07	7188.29	1917.77	1	189.3
H(3c2)	120.72	7777.96	2122.38	1	189.3
H(1c1b)	6016.79	1139.95	202.91	1	329.7
H(1c1c)	5866.28	4182.48	314.72	1	329.7

H(2c1c)	7413.48	3365.72	334.13	1	329.7
H(3c1c)	6565.99	3433.88	65.56	1	329.7
H(1c1d)	3678.53	1501.53	137.03	1	329.7
H(2c1d)	3499.75	1822.14	437.31	1	329.7
H(3c1d)	3797.03	3144.03	242.42	1	329.7
H(1n1)	4909.86	2915.42	824.77	1	329.7
H(2n1)	3893.96	2038.01	675.89	1	329.7
H(3n1)	4615.75	1411.33	898.2	1	329.7
H(1n2)	656.55	5748.83	1780.62	1	189.3
H(2n2)	1514.56	6993.92	1695.21	1	189.3
H(3n2)	1751.42	6384.03	1948.82	1	189.3

*U_{eq} is defined as one third of the trace of the orthogonalized U_{ij} tensor.

Table S8. Anisotropic displacement parameters (Å^2 x 10^3) for IBA n = 3 at 293 K with estimated standard deviations in parentheses.

Label	U_{11}	U_{22}	U_{33}	U_{12}	U_{13}	U_{23}
Pb(1)	62(2)	42(2)	51(2)	10(2)	2(1)	2(3)
Pb(2)	52(2)	40(2)	52(2)	0	0(2)	0
I(1)	97(4)	92(4)	234(6)	56(3)	3(4)	3(4)
I(2)	78(4)	78(6)	254(14)	62(4)	-10(6)	4(5)
I(3)	76(5)	75(6)	280(15)	-6(4)	8(6)	10(6)
I(4)	234(6)	264(7)	36(3)	-30(12)	3(3)	-5(11)
I(5)	359(11)	434(13)	39(4)	-62(15)	-5(5)	-17(14)

The anisotropic displacement factor exponent takes the form: $-2\pi^2[h^2a^{*2}U_{11} + ... + 2hka^*b^*U_{12}]$.

Table S9. Selected bonds and angles for IBA n = 3 at 293 K with estimated standard deviations in parentheses.

Label	Distances (Å)	Label	Angles (°)
Pb(1)-I(2)	3.214(6)	Pb(2)-I(1)-Pb(2)#5	177.2(3)
Pb(1)-I(2)#1	3.111(7)	Pb(1)-I(2)-Pb(1)#5	176.6(3)
Pb(1)-I(3)	3.108(7)	Pb(1)-I(3)-Pb(1)#6	176.8(3)
Pb(1)-I(3)#2	3.218(7)	Pb(1)-I(4)-Pb(2)	176.7(5)
Pb(1)-I(4)	3.268(5)		
Pb(1)-I(5)	3.019(6)		
Pb(2)-I(1)	3.201(10)		
Pb(2)-I(1)#1	3.124(10)		
Pb(2)-I(1)#3	3.201(10)		
Pb(2)-I(1)#4	3.124(10)		
Pb(2)-I(4)	3.129(5)		
Pb(2)-I(4)#3	3.129(5)		

Table S10. Atomic coordinates (x10^4) and equivalent isotropic displacement parameters (Å^2 x 10^3) for IBA n = 4 at 293 K with estimated standard deviations in parentheses.

Label	x	y	z	Occupancy	U_{eq}*
Pb(1)	4992(5)	2465(5)	2003(1)	1	59(2)
Pb(2)	4996(4)	2459(6)	1000(1)	1	60(2)
I(1)	2512(13)	-6(10)	1012(3)	1	144(10)
I(2)	7483(16)	-6(10)	1014(3)	1	159(11)
I(3)	2505(16)	7(12)	1999(3)	1	147(11)
I(4)	4964(10)	2294(15)	1515(1)	1	175(7)
I(5)	2503(14)	5007(12)	2006(3)	1	137(10)
I(6)	5000	2280(20)	2500	1	184(11)
I(7)	4960(13)	1979(11)	524(1)	1	202(8)
N(1)	5000	-1860(60)	2500	1	90(20)
C(2b)	4970(40)	-1460(40)	142(5)	1	107(14)
C(2c)	5360(60)	-2180(70)	-63(5)	1	107(14)
N(4)	4270(50)	-1710(50)	1473(6)	1	55(11)
C(4)	5830(40)	-1970(50)	1551(8)	1	55(11)
C(2a)	5650(40)	-2330(50)	319(5)	1	107(14)
N(2)	5000(70)	-1810(80)	524(5)	1	107(14)
C(2d)	3300(40)	-1440(70)	167(7)	1	107(14)
C(1)	5000	-190(60)	2500	1	90(20)
H(1c2b)	5340.29	-451.19	143.87	1	128.4
H(1c2c)	6198.95	-1672.93	-124.56	1	128.4

H(2c2c)	5615.24	-3211.95	-39.55	1	128.4
H(3c2c)	4520.31	-2125.56	-156.8	1	128.4
H(1c4)	5840.19	-2841.62	1639.8	1	65.7
H(2c4)	6486.56	-2134.54	1433.52	1	65.7
H(3c4)	6163.81	-1119.58	1629.19	1	65.7
H(1c2a)	5449.68	-3373.17	300.17	1	128.4
H(2c2a)	6716.12	-2168.12	319.85	1	128.4
H(1c2d)	2876.33	-696.32	75.48	1	128.4
H(2c2d)	2898.68	-2402.19	132.59	1	128.4
H(3c2d)	3051.37	-1197.04	310.25	1	128.4
H(1c1)	4040.12	171.55	2545.19	0.5	104.1
H(2c1)	5758.8	171.55	2594.17	0.5	104.1
H(3c1)	5201.08	171.55	2360.64	0.5	104.1
H(1n1)	4114.91	-2188.97	2466	0.5	104.1
H(2n1)	5651.97	-2188.97	2409.35	0.5	104.1
H(3n1)	5233.12	-2188.97	2624.65	0.5	104.1
H(1n4)	3666.25	-2351.86	1529.37	1	65.7
H(2n4)	4250.76	-1804.99	1336.56	1	65.7
H(1n2)	5270.03	-2421.73	623.22	1	128.4
H(2n2)	5327.62	-916.48	552.01	1	128.4
H(3n2)	4031.57	-1796.33	514.31	1	128.4

*U_{eq} is defined as one third of the trace of the orthogonalized U_{ij} tensor.

Table S11. Anisotropic displacement parameters (Å^2 x 10^3) for IBA n = 4 at 293 K with estimated standard deviations in parentheses.

Label	U_{11}	U_{22}	U_{33}	U_{12}	U_{13}	U_{23}
Pb(1)	70(4)	39(3)	67(2)	0(5)	-14(2)	3(4)
Pb(2)	67(5)	44(4)	70(2)	3(4)	-11(2)	8(3)
I(1)	76(16)	59(12)	300(20)	-14(7)	-19(15)	-4(7)
I(2)	140(20)	68(12)	270(20)	54(8)	-18(17)	1(8)
I(3)	122(17)	76(15)	240(20)	-49(8)	-11(16)	7(7)
I(4)	260(15)	232(15)	35(4)	-6(18)	10(5)	5(9)
I(5)	80(14)	66(14)	270(20)	26(7)	-20(14)	4(7)
I(6)	280(20)	220(20)	52(7)	0	8(9)	0
I(7)	410(20)	109(10)	82(6)	-15(8)	-15(9)	-2(4)

The anisotropic displacement factor exponent takes the form: $-2\pi^2[h^2a^{*2}U_{11} + ... + 2hka^*b^*U_{12}]$.

Table S12. Selected bonds and angles for IBA n = 4 at 293 K with estimated standard deviations in parentheses.

Label	Distances (Å)	Label	Angles (°)
Pb(1)-I(3)	3.126(14)	Pb(2)-I(1)-Pb(2)#4	177.3(6)
Pb(1)-I(3)#1	3.195(14)	Pb(2)-I(2)-Pb(2)#2	176.8(6)
Pb(1)-I(4)	3.110(6)	Pb(1)-I(3)-Pb(1)#4	178.9(6)
Pb(1)-I(5)	3.181(13)	Pb(1)-I(4)-Pb(2)	174.5(5)
Pb(1)-I(5)#2	3.140(13)	Pb(1)-I(5)-Pb(1)#3	178.6(5)
Pb(1)-I(6)	3.169(3)	Pb(1)-I(6)-Pb(1)#5	174.1(8)
Pb(2)-I(1)	3.129(11)		
Pb(2)-I(1)#1	3.194(11)		
Pb(2)-I(2)	3.131(13)		
Pb(2)-I(2)#3	3.192(13)		
Pb(2)-I(4)	3.277(6)		
Pb(2)-I(7)	3.061(6)		

Table S13. Atomic coordinates (x10^4) and equivalent isotropic displacement parameters (Å^2 x 10^3) for IAA n = 1 at 293 K with estimated standard deviations in parentheses.

Label	x	y	z	Occupancy	U_{eq}^*
Pb(1)	5000	5000	0	1	92(2)
I(1)	7020(2)	4996(5)	1021(6)	1	143(2)
I(2)	5003(4)	1889(3)	1911(4)	1	122(2)
N(1)	3540(30)	-830(60)	130(70)	1	293(16)
C(1b)	2330(20)	-250(60)	-2110(50)	1	293(16)
C(1a)	3070(30)	410(50)	-920(70)	1	293(16)
C(1d)	1410(30)	-540(80)	-360(60)	1	293(16)
C(1c)	1540(20)	290(40)	-1770(50)	1	293(16)
C(1e)	820(20)	0(90)	-3180(60)	1	293(16)
H(1c1b)	2358.51	-1348.2	-2039.82	1	351.1
H(2c1b)	2344.49	60.14	-3149.07	1	351.1
H(1c1a)	2881.34	1155.35	-285.39	1	351.1
H(2c1a)	3438.22	894.42	-1455.98	1	351.1
H(1c1d)	878.74	-243.87	-194.43	1	351.1
H(2c1d)	1403.82	-1621.49	-553.86	1	351.1
H(3c1d)	1849.33	-296.33	554.6	1	351.1
H(1c1c)	1581.81	1368.49	-1551.1	1	351.1
H(1c1e)	304.87	-6.15	-2859.55	1	351.1
H(2c1e)	791.16	785.74	-3952.7	1	351.1
H(3c1e)	888.34	-973.64	-3632.02	1	351.1

H(1n1)	3940.04	-435.81	868.47	1	351.1
H(2n1)	3193.93	-1316.65	560.97	1	351.1
H(3n1)	3743.82	-1465.48	-429.01	1	351.1

*U_{eq} is defined as one third of the trace of the orthogonalized U_{ij} tensor.

Table S14. Anisotropic displacement parameters (Å^2 x 10^3) for IAA n = 1 at 293 K with estimated standard deviations in parentheses.

Label	U_{11}	U_{22}	U_{33}	U_{12}	U_{13}	U_{23}
Pb(1)	188(2)	34(2)	68(2)	-1(3)	58(2)	0(2)
I(1)	168(2)	122(4)	161(5)	4(4)	83(4)	8(3)
I(2)	255(3)	58(2)	76(2)	1(3)	84(3)	22(2)

The anisotropic displacement factor exponent takes the form: $-2\pi^2[h^2a^{*2}U_{11} + ... + 2hka^*b^*U_{12}]$.

Table S15. Selected bonds and angles for IAA n = 1 at 293 K with estimated standard deviations in parentheses.

Label	Distances (Å)	Label	Angles (°)
Pb(1)-I(1)	3.226(2)	Pb(1)-I(2)-Pb(1)#5	152.97(11)
Pb(1)-I(1)#1	3.226(2)		
Pb(1)-I(2)	3.199(4)		
Pb(1)-I(2)#2	3.176(3)		
Pb(1)-I(2)#1	3.199(4)		
Pb(1)-I(2)#3	3.176(3)		

Table S16. Atomic coordinates (x 10^4), occupancy and equivalent isotropic displacement parameters for IAA n = 2 at 273 K with estimated standard deviations in parentheses.

Label	x	y	z	Occupancy	U_{eq}*
Pb(1)	7500(4)	-2918(3)	-2501(4)	1	89(4)
Pb(2)	2509(4)	-7927(3)	-2503(4)	1	71(3)
Pb(3)	7495(5)	-1402(3)	-2497(4)	1	76(3)
Pb(4)	2504(4)	-6410(3)	-2489(4)	1	66(3)
I(1)	0	-2919(5)	-5000	1	148(9)
I(2)	5000	-2877(5)	0	1	144(9)
I(3)	0	-2854(4)	0	1	124(8)
I(4)	5000	-2920(6)	-5000	1	390(20)
I(5)	7441(8)	-2162(4)	-2638(7)	1	182(5)
I(6)	0	-7801(3)	-5000	1	84(6)
I(7)	5000	-8037(4)	0	1	143(8)
I(8)	7628(12)	-3644(4)	-2231(11)	1	217(8)
I(9)	7545(14)	-701(4)	-2344(12)	1	272(9)
I(10)	5000	-1416(4)	0	1	127(8)
I(11)	0	-7945(5)	0	1	152(10)
I(12)	0	-1392(5)	-5000	1	150(9)
I(13)	2836(6)	-7170(4)	-2122(5)	1	79(3)
I(15)	5000	-7895(4)	-5000	1	104(8)
I(18)	2124(9)	-8638(4)	-2994(8)	1	91(5)
I(20)	0	-6435(4)	0	1	93(7)

I(21)	5000	-6426(4)	-5000	1	143(9)
I(22)	10000	-1474(6)	0	1	349(18)
I(23)	5000	-1401(4)	-5000	1	130(8)
I(24)	2024(9)	-5697(3)	-2950(9)	1	98(5)
I(25)	5000	-6316(4)	0	1	99(7)
I(26)	0	-10000(200)	-5000	1	97(6)

*U_{eq} is defined as one third of the trace of the orthogonalized U_{ij} tensor.

Table S17. Anisotropic displacement parameters (Å^2 x 10^3) for IAA n = 2 at 273 K with estimated standard deviations in parentheses.

Label	U_{11}	U_{22}	U_{33}	U_{12}	U_{13}	U_{23}
Pb(1)	84(6)	133(8)	52(5)	-2(3)	-33(4)	-11(3)
Pb(2)	23(5)	103(7)	86(5)	-3(3)	6(4)	5(3)
Pb(3)	105(6)	112(7)	11(4)	3(3)	-10(4)	-11(3)
Pb(4)	48(5)	86(7)	63(5)	1(3)	25(4)	13(3)
I(1)	133(14)	200(20)	109(13)	0	29(10)	0
I(2)	187(16)	130(16)	114(13)	0	22(11)	0
I(3)	120(12)	217(17)	38(10)	0	-66(8)	0
I(4)	200(20)	820(50)	137(18)	0	-122(14)	0
I(5)	252(9)	129(10)	164(8)	62(10)	38(6)	74(10)
I(6)	14(8)	89(11)	149(12)	0	-48(7)	0
I(7)	125(13)	143(16)	161(15)	0	-47(10)	0
I(8)	305(15)	139(13)	206(12)	-92(9)	31(10)	-62(9)
I(9)	390(19)	139(13)	286(15)	-120(10)	102(13)	-70(10)
I(10)	129(13)	179(18)	73(11)	0	49(9)	0
I(11)	132(14)	250(20)	79(12)	0	60(10)	0
I(12)	169(15)	178(19)	101(12)	0	52(10)	0
I(13)	93(5)	59(7)	83(5)	8(7)	-16(3)	-40(7)
I(15)	33(10)	125(16)	153(13)	0	67(9)	0
I(18)	117(8)	81(10)	75(7)	-9(6)	-34(5)	1(6)
I(20)	69(11)	111(15)	97(11)	0	77(8)	0

I(21)	119(14)	200(20)	113(12)	0	76(10)	0
I(22)	230(20)	700(50)	119(17)	0	-120(14)	0
I(23)	142(13)	242(19)	8(10)	0	-65(8)	0
I(24)	108(8)	65(9)	121(8)	3(6)	-40(6)	15(6)
I(25)	61(10)	96(14)	142(12)	0	-78(8)	0
I(26)	144(12)	92(11)	55(10)	0	-37(7)	0

Table S18. Selected bond and angle distributions for IAA n = 2 at 293 K with estimated standard deviations in parentheses.

Label	Average Distance	Minimum Distance	Maximum Distance
Pb(1)-I(1)#1	3.172(15)	3.159(19)	3.185(19)
Pb(1)-I(2)	3.185(14)	3.167(18)	3.203(18)
Pb(1)-I(3)#1	3.149(14)	3.11(2)	3.19(2)
Pb(1)-I(4)	3.191(15)	3.14(2)	3.24(2)
Pb(1)-I(5)	3.26(3)	3.24(3)	3.27(3)
Pb(1)-I(8)	3.13(3)	3.12(3)	3.15(3)
Pb(2)-I(6)	3.184(16)	3.15(2)	3.21(2)
Pb(2)-I(7)	3.155(18)	3.00(2)	3.31(2)
Pb(2)-I(11)	3.141(18)	3.11(3)	3.17(3)
Pb(2)-I(13)	3.26(3)	3.25(3)	3.28(3)
Pb(2)-I(15)	3.135(16)	3.12(2)	3.15(2)
Pb(2)-I(18)	3.11(3)	3.06(3)	3.15(3)
Pb(3)-I(10)	3.204(15)	3.173(19)	3.235(19)
Pb(3)-I(12)#1	3.144(15)	3.13(2)	3.16(2)
Pb(3)-I(22)	3.181(15)	3.15(2)	3.22(2)
Pb(3)-I(23)	3.157(14)	3.09(2)	3.23(2)
Pb(4)-I(13)	3.22(3)	3.20(3)	3.25(3)
Pb(4)-I(20)	3.119(16)	3.10(2)	3.14(2)
Pb(4)-I(21)	3.150(18)	3.13(3)	3.17(3)
Pb(4)-I(24)	3.04(3)	2.98(3)	3.10(3)
Pb(4)-I(25)	3.188(16)	3.13(2)	3.25(2)
Pb(4)-I(26)	3(3)	3(7)	3(7)
**Label**	**Average Angle**	**Minimun Angle**	**Maximum Angle**
Pb(1)#2-I(1)-Pb(1)#3	160.1(10)	158.9(11)	161.5(11)

Pb(1)-I(2)-Pb(1)#4	159.1(10)	157.2(10)	161.2(11)
Pb(1)#2-I(3)-Pb(1)#4	174.6(9)	173.7(8)	176.0(12)
Pb(1)-I(4)-Pb(1)#3	175.4(8)	174.7(8)	177.4(13)
Pb(1)-I(5)-Pb(3)	164.1(6)	163.4(6)	164.9(6)
Pb(2)-I(6)-Pb(2)#5	174.4(9)	169.9(10)	178.9(9)
Pb(2)-I(7)-Pb(2)#4	177.4(8)	176.5(9)	178.8(3)
Pb(3)-I(10)-Pb(3)#4	156.4(10)	153.5(11)	159.4(11)
Pb(2)-I(11)-Pb(2)#6	174.4(13)	171.3(17)	177.0(10)
Pb(3)#2-I(12)-Pb(3)#3	166.2(10)	162.7(11)	170.4(11)
Pb(2)-I(13)-Pb(4)	174.8(9)	173.1(8)	176.5(8)
Pb(2)-I(15)-Pb(2)#3	168.5(11)	166.7(14)	170.9(14)
Pb(4)-I(20)-Pb(4)#6	177.0(10)	176.7(14)	177.3(9)
Pb(4)-I(21)-Pb(4)#3	170.3(12)	166.9(16)	174.6(16)
Pb(3)-I(22)-Pb(3)#7	176.3(8)	175.0(8)	179.8(13)
Pb(3)-I(23)-Pb(3)#3	175.6(10)	174.9(10)	177.3(13)
Pb(4)-I(25)-Pb(4)#4	177.9(9)	176.5(11)	179.0(7)
Pb(4)-I(26)-Pb(4)#5	0(3000)	0(3000)	0(3000)

Table S19. Atomic coordinates (x10^4) and equivalent isotropic displacement parameters (Å^2 x 10^3) for IAA n = 3 at 293 K with estimated standard deviations in parentheses.

Label	x	y	z	Occupancy	U_{eq}^*
Pb(1)	7500	2500	0	1	60(1)
Pb(2)	7512(3)	1338(1)	12(3)	1	56(1)
I(1)	5000	1336(2)	2500	1	151(4)
I(2)	0	1333(2)	2500	1	144(4)
I(3)	7547(7)	1933(1)	194(5)	1	105(2)
I(4)	5000	3612(2)	2500	1	126(4)
I(5)	0	3610(2)	2500	1	126(4)
I(6)	7428(9)	4203(1)	327(6)	1	180(3)
I(7)	0	2503(2)	2500	1	261(4)
I(8)	5000	2518(2)	2500	1	266(4)
N(1)	7940(70)	3990(9)	-5090(80)	1	360(20)
C(1a)	7220(60)	4211(10)	-4440(70)	1	360(20)
C(1b)	8350(70)	4410(9)	-4380(70)	1	360(20)
C(1c)	7720(60)	4629(9)	-5140(40)	1	360(20)
N(2)	-2090(70)	3080(14)	4570(60)	1	270(20)
C(2)	-2220(100)	3035(11)	6230(60)	1	270(20)
C(1d)	8530(80)	4848(9)	-4590(80)	1	360(20)
C(1e)	6080(60)	4651(13)	-4780(70)	1	360(20)
H(1c1a)	6865.75	4176.12	-3445.94	1	436.2
H(2c1a)	6390.34	4258.07	-5062.52	1	436.2

H(1c1b)	8569.73	4447.36	-3345.94	1	436.2
H(2c1b)	9251.98	4361.35	-4876.79	1	436.2
H(1c1c)	7844.44	4613.76	-6203.47	1	436.2
H(1c2)	-2604.3	2876.13	6394.06	1	319.7
H(2c2)	-1245.1	3048.86	6685.94	1	319.7
H(3c2)	-2886.17	3150.62	6663.89	1	319.7
H(1c1d)	8186.72	4888.38	-3602.44	1	436.2
H(2c1d)	9591.24	4816.05	-4561.23	1	436.2
H(3c1d)	8339	4979.25	-5261.96	1	436.2
H(1c1e)	5532.78	4689.28	-5673.32	1	436.2
H(2c1e)	5720.27	4501.39	-4375.37	1	436.2
H(3c1e)	5938.02	4775.76	-4046.97	1	436.2
H(1n1)	7285.72	3875.04	-5134.7	1	436.2
H(2n1)	8270.54	4022.09	-5988.08	1	436.2
H(3n1)	8689.31	3946.72	-4521.79	1	436.2
H(1n2)	-2956.21	3123.76	4218.43	1	319.7
H(2n2)	-1433.09	3193.01	4413.16	1	319.7
H(3n2)	-1798.13	2948.98	4124.37	1	319.7

*U_{eq} is defined as one third of the trace of the orthogonalized U_{ij} tensor.

Table S20. Anisotropic displacement parameters (Å^2 x 10^3) for IAA n = 3 at 293 K with estimated standard deviations in parentheses.

Label	U_{11}	U_{22}	U_{33}	U_{12}	U_{13}	U_{23}
Pb(1)	52(2)	78(2)	50(2)	7(3)	-2(1)	-5(2)
Pb(2)	40(1)	88(2)	41(1)	1(2)	-3(1)	2(2)
I(1)	90(7)	272(10)	90(5)	0	41(5)	0
I(2)	97(6)	248(10)	86(5)	0	-57(4)	0
I(3)	134(2)	73(2)	108(3)	-9(4)	-12(2)	6(3)
I(4)	90(6)	200(8)	90(5)	0	50(4)	0
I(5)	86(6)	192(8)	101(5)	0	-55(4)	0
I(6)	272(6)	89(3)	180(5)	1(5)	17(5)	-5(3)
I(7)	314(8)	140(5)	329(9)	0	-298(7)	0
I(8)	322(9)	151(6)	325(9)	0	299(8)	0

The anisotropic displacement factor exponent takes the form: $-2\pi^2[h^2a^{*2}U_{11} + ... + 2hka^*b^*U_{12}]$.

Table S21. Selected bonds and angles for IAA n = 3 at 293 K with estimated standard deviations in parentheses.

Label	Distances (Å)	Label	Angles (°)
Pb(1)-I(3)	3.160(3)	Pb(2)-I(1)-Pb(2)#7	179.6(3)
Pb(1)-I(3)#1	3.160(3)	Pb(2)#8-I(2)-Pb(2)#7	178.8(3)
Pb(1)-I(7)#2	3.1467(11)	Pb(1)-I(3)-Pb(2)	173.93(15)
Pb(1)-I(7)#3	3.1467(11)	Pb(2)#1-I(4)-Pb(2)#4	170.1(2)
Pb(1)-I(8)	3.1513(12)	Pb(2)#3-I(5)-Pb(2)#4	169.6(3)
Pb(1)-I(8)#1	3.1513(12)	Pb(1)#8-I(7)-Pb(1)#7	179.3(4)
Pb(2)-I(1)	3.150(3)	Pb(1)-I(8)-Pb(1)#7	176.3(4)
Pb(2)-I(2)#2	3.132(3)		
Pb(2)-I(3)	3.317(3)		
Pb(2)-I(4)#1	3.161(3)		
Pb(2)-I(5)#3	3.175(3)		
Pb(2)-I(6)#1	3.030(4)		

Table S22. Atomic coordinates (x10^4) and equivalent isotropic displacement parameters (Å^2 x 10^3) for IAA n = 4 at 293 K with estimated standard deviations in parentheses.

Label	x	y	z	Occupancy	U_{eq}^*
Pb(1)	2487(7)	468(1)	2493(7)	1	72(2)
Pb(2)	2511(6)	1409(1)	2497(5)	1	74(2)
I(1)	2872(12)	923(2)	2748(11)	1	125(4)
I(2)	2350(20)	0	2123(10)	1	149(7)
I(3)	5000	1397(3)	0	1	148(9)
I(4)	5000	1442(3)	5000	1	147(8)
I(5)	0	1341(2)	0	1	136(7)
I(6)	0	1394(3)	5000	1	131(7)
I(7)	0	519(3)	0	1	166(9)
I(8)	2078(16)	1849(2)	2184(15)	1	175(7)
I(9)	0	470(3)	5000	1	134(8)
I(10)	5000	466(3)	0	1	200(11)
I(11)	5000	418(3)	5000	1	158(9)
N(2)	3980(60)	0	5710(70)	1	58(17)
N(3)	-430(80)	929(12)	1770(70)	1	150(20)
N(4)	2140(130)	1699(11)	-3180(110)	1	220(30)
C(3a)	-1800(80)	917(13)	2720(90)	1	150(20)
C(2a)	2880(70)	0	6970(70)	1	58(17)
C(4a)	2270(150)	1832(11)	-1850(100)	1	220(30)
C(4b)	3350(80)	1994(10)	-2170(120)	1	220(30)

C(4c)	2640(90)	2188(10)	-1830(70)	1	220(30)
C(4d)	3670(130)	2306(11)	-850(80)	1	220(30)
C(4e)	2350(150)	2297(13)	-3270(40)	1	220(30)
H(1c3a)	-2093.22	782.32	2835.65	1	185.2
H(2c3a)	-1591.35	972.84	3688.61	1	185.2
H(3c3a)	-2605.93	988.77	2248.59	1	185.2
H(1c2a)	2661.26	-132.78	7256.63	0.5	69.8
H(2c2a)	3290.14	69.66	7811.95	0.5	69.8
H(3c2a)	1967.21	63.12	6643.76	0.5	69.8
H(1c4a)	2614.33	1758.47	-994.65	1	263.6
H(2c4a)	1300.11	1887.06	-1635.37	1	263.6
H(1c4b)	4233.39	1978	-1563.86	1	263.6
H(2c4b)	3618.43	1990	-3214.51	1	263.6
H(1c4c)	1708.39	2166.92	-1323.26	1	263.6
H(1c4d)	4675.45	2295.09	-1206.08	1	263.6
H(2c4d)	3367.45	2441.64	-869.31	1	263.6
H(3c4d)	3622.89	2258.16	162.92	1	263.6
H(1c4e)	3198.47	2285.29	-3907.67	1	263.6
H(2c4e)	1480.02	2243	-3760.98	1	263.6
H(3c4e)	2171.24	2433.3	-3048.94	1	263.6
H(1n2)	4483.89	-109.25	5725.22	0.5	69.8
H(2n2)	3503.5	10.85	4859.5	0.5	69.8
H(3n2)	4592.04	98.4	5820.72	0.5	69.8

H(1n3)	81.93	821.11	1860.02	1	185.2
H(2n3)	-698.14	944.34	831.52	1	185.2
H(3n3)	113.44	1028.05	2051.78	1	185.2
H(1n4)	2459.58	1582.38	-2940.05	1	263.6
H(2n4)	1211.56	1692.09	-3476.2	1	263.6
H(3n4)	2684.81	1745.2	-3907.48	1	263.6

*U_{eq} is defined as one third of the trace of the orthogonalized U_{ij} tensor.

Table S23. Anisotropic displacement parameters ($Å^2$ x 10^3) for IAA n = 4 at 293 K with estimated standard deviations in parentheses.

Label	U_{11}	U_{22}	U_{33}	U_{12}	U_{13}	U_{23}
Pb(1)	73(2)	85(3)	59(2)	-5(3)	-15(2)	1(3)
Pb(2)	77(2)	91(3)	52(2)	4(3)	-8(2)	0(3)
I(1)	167(10)	70(6)	136(7)	-16(7)	-10(6)	-2(6)
I(2)	342(18)	43(7)	62(7)	0	58(10)	0
I(3)	115(12)	240(20)	93(9)	0	35(8)	0
I(4)	113(10)	225(18)	103(8)	0	-60(8)	0
I(5)	109(9)	189(16)	108(9)	0	-68(8)	0
I(6)	94(10)	209(18)	90(8)	0	-17(7)	0
I(7)	141(12)	203(19)	151(14)	0	-106(11)	0
I(8)	226(14)	86(8)	212(12)	-8(7)	-70(9)	26(7)
I(9)	86(7)	190(20)	125(11)	0	66(7)	0
I(10)	340(20)	143(19)	119(12)	0	92(13)	0
I(11)	178(15)	162(17)	132(13)	0	-98(11)	0

The anisotropic displacement factor exponent takes the form: $-2\pi^2[h^2a^{*2}U_{11} + ... + 2hka^*b^*U_{12}]$.

Table S24. Selected bonds and angles for IAA n = 4 at 293 K with estimated standard deviations in parentheses.

Label	Distances (Å)	Label	Angles (°)
Pb(1)-I(1)	3.127(10)	Pb(1)-I(1)-Pb(2)	165.7(4)
Pb(1)-I(2)	3.208(5)	Pb(1)-I(2)-Pb(1)#5	167.4(4)
Pb(1)-I(7)	3.146(6)	Pb(2)-I(3)-Pb(2)#3	177.2(7)
Pb(1)-I(9)	3.166(6)	Pb(2)-I(4)-Pb(2)#1	171.8(7)
Pb(1)-I(10)	3.172(6)	Pb(2)-I(5)-Pb(2)#4	163.3(6)
Pb(1)-I(11)	3.170(6)	Pb(2)-I(6)-Pb(2)#6	176.5(6)
Pb(2)-I(1)	3.334(9)	Pb(1)-I(7)-Pb(1)#4	167.2(7)
Pb(2)-I(3)	3.161(5)	Pb(1)-I(9)-Pb(1)#6	179.6(7)
Pb(2)-I(4)	3.142(5)	Pb(1)-I(10)-Pb(1)#3	179.6(7)
Pb(2)-I(5)	3.177(6)	Pb(1)-I(11)-Pb(1)#1	167.6(6)
Pb(2)-I(6)	3.180(5)		
Pb(2)-I(8)	3.037(9)		

Table S25. Optical data for both systems.

Compound	E_g	E_{exc}	PL	VBM	CBM
IBA n = 1	2.41	2.35	2.36	-5.6	-3.14
IBA n = 2	2.19	2.14	2.15	-5.54	-3.35
IBA n = 3	1.98	1.95	1.99	-5.43	-3.45
IBA n = 4	1.90	1.85	1.89	-5.44	-3.54
IAA n = 1	2.46	2.41	2.40	-5.63	-3.17
IAA n = 2	2.15	2.12	2.14	-5.56	-3.41
IAA n = 3	2.02	1.98	2.02	-5.54	-3.52
IAA n = 4	1.91	1.88	1.91	-5.50	-3.59

Table S26. Statistics of Solar Cell Devices for IBA and IAA n = 4.

Compound	V_{OC} (V)	J_{SC} (mA·cm^{-2})	FF (%)	PCE (%)
IBA n = 4	0.914 ± 0.009	11.38 ± 0.21	71.6 ± 4.7	7.45 ± 0.53
IAA n = 4	0.999 ± 0.015	9.43 ± 0.76	65.0 ± 4.8	6.13 ± 0.73

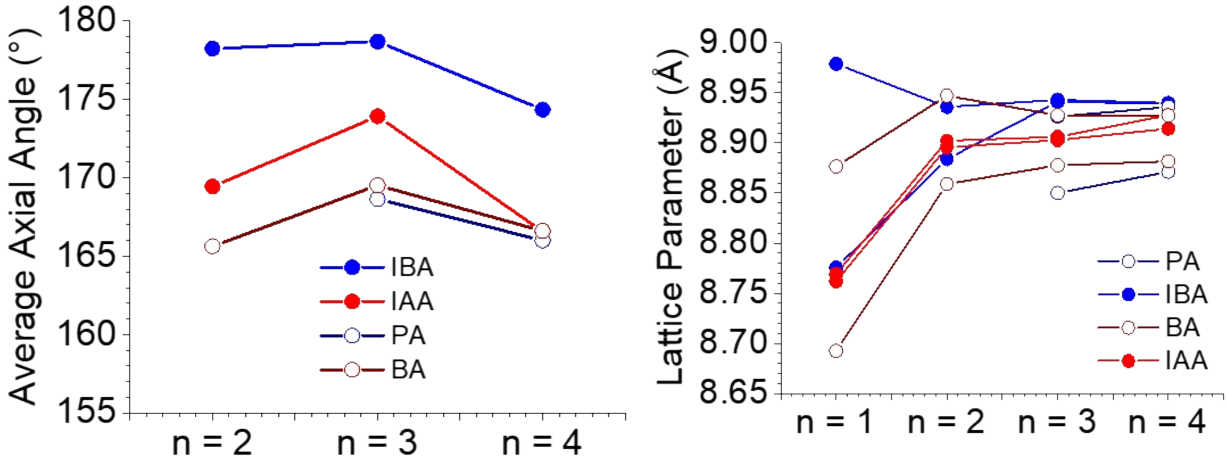

Figure S1. Out-of-plane angles (left) and lattice parameters (right) for branched and straight-chain alkylamine perovskites.

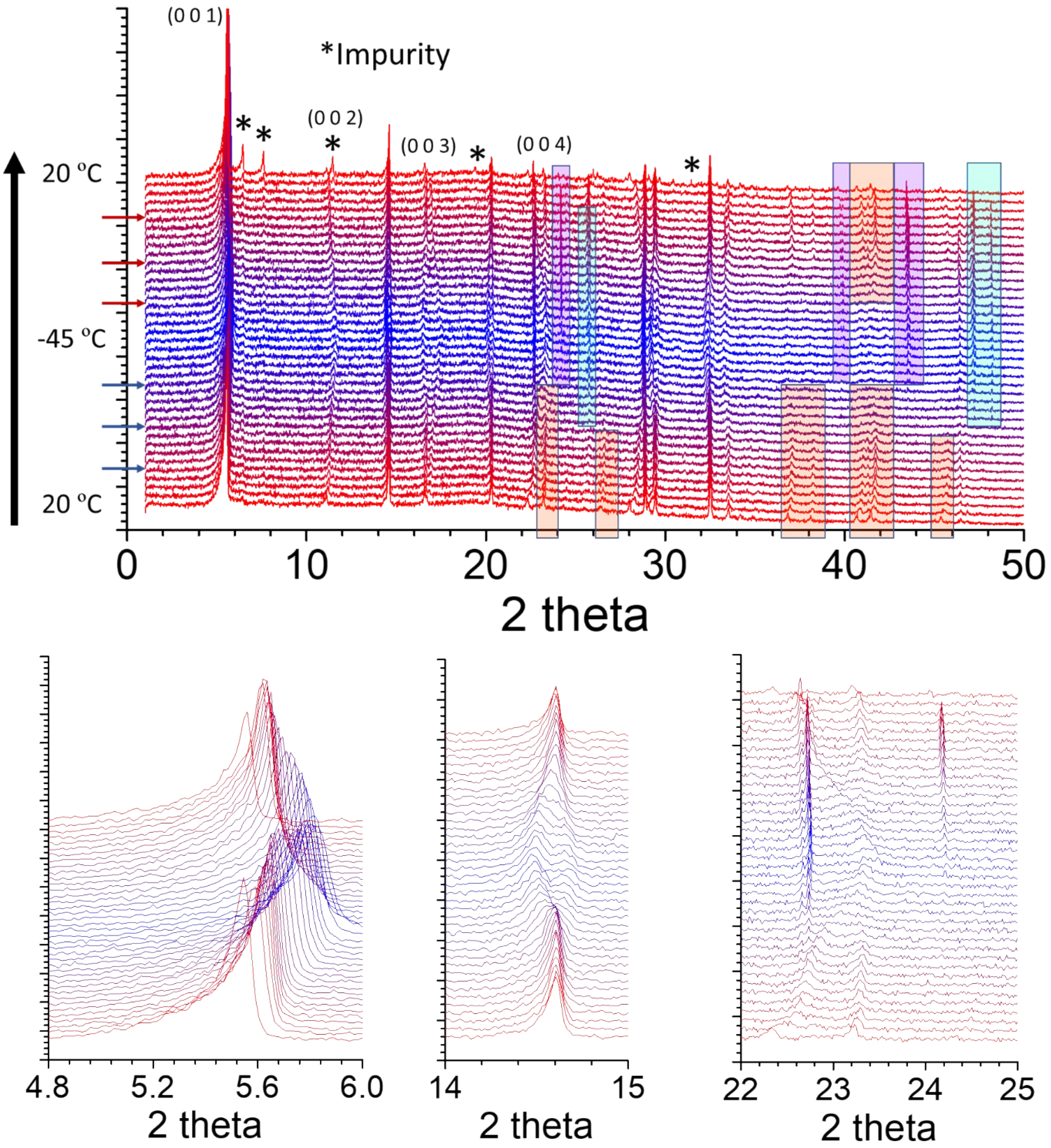

Figure S2. Variable-temperature PXRD of IAA n = 1, showing the several phase transitions which appeared in the DSC measurements. Beginning from the bottom of the top figure, peaks highlighted in orange disappear between -20 °C and -30 °C. Blue peaks grow in simultaneously, corresponding to the broad peak in this region of the DSC. Purple peaks grow in at -30 °C, directly corresponding to the sharp transition in DSC. Below are enlarged areas of the above graph. The left shows a large contraction of the (0 0 1) peak at low temperature, showing the decrease in interlayer spacing. The middle shows the (1 1 0) and (-1 1 1) peaks separating at symmetry is broken. The right shows several peaks disappearing and appearing at low temperatures.

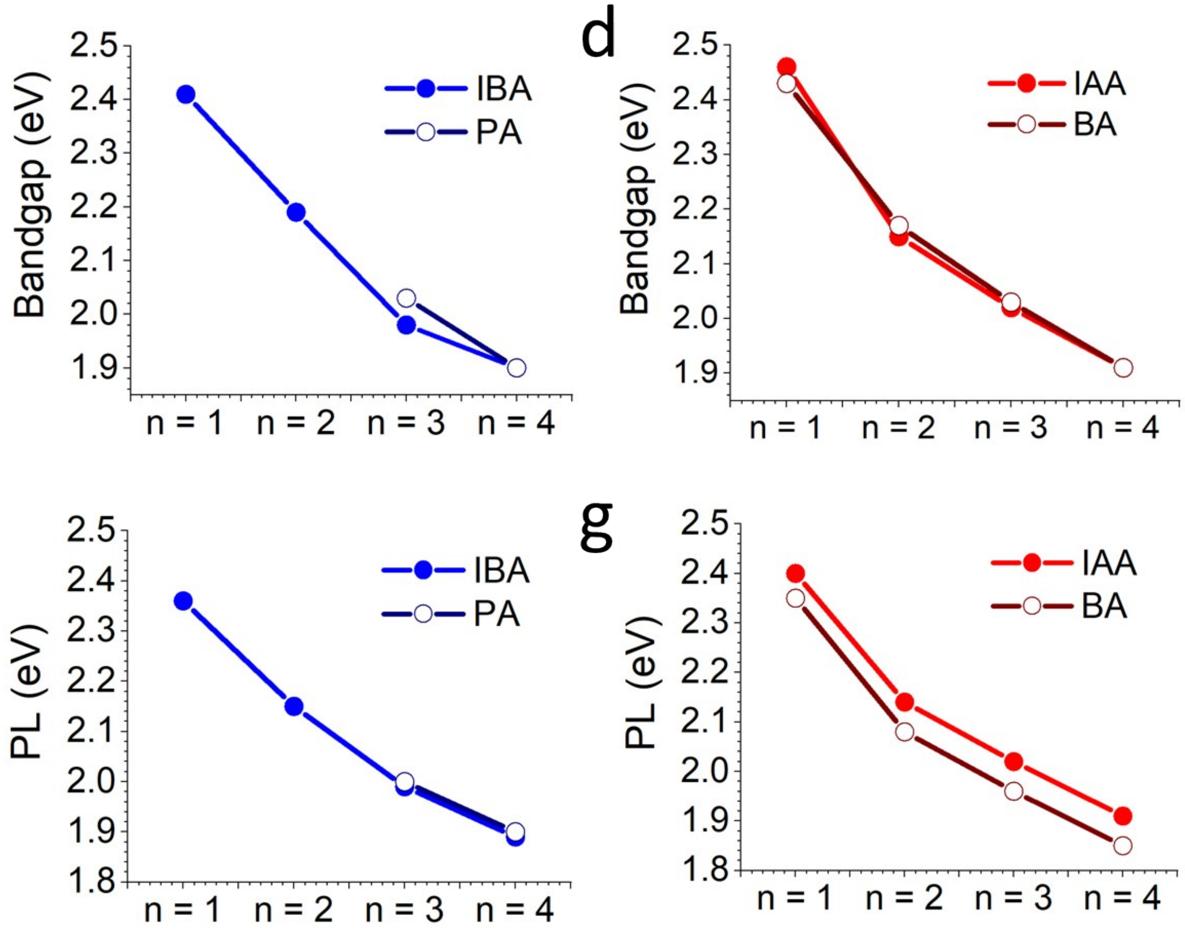

Figure S3. (a,b) A comparison of the bandgaps for branched and straight spacers showing a slight red shift for the branched systems and (c,d) a comparison of the PL, showing IAA to be blue-shifted for all n values and IBA to be similar to that of PA, which is unexpected based on bond angles.

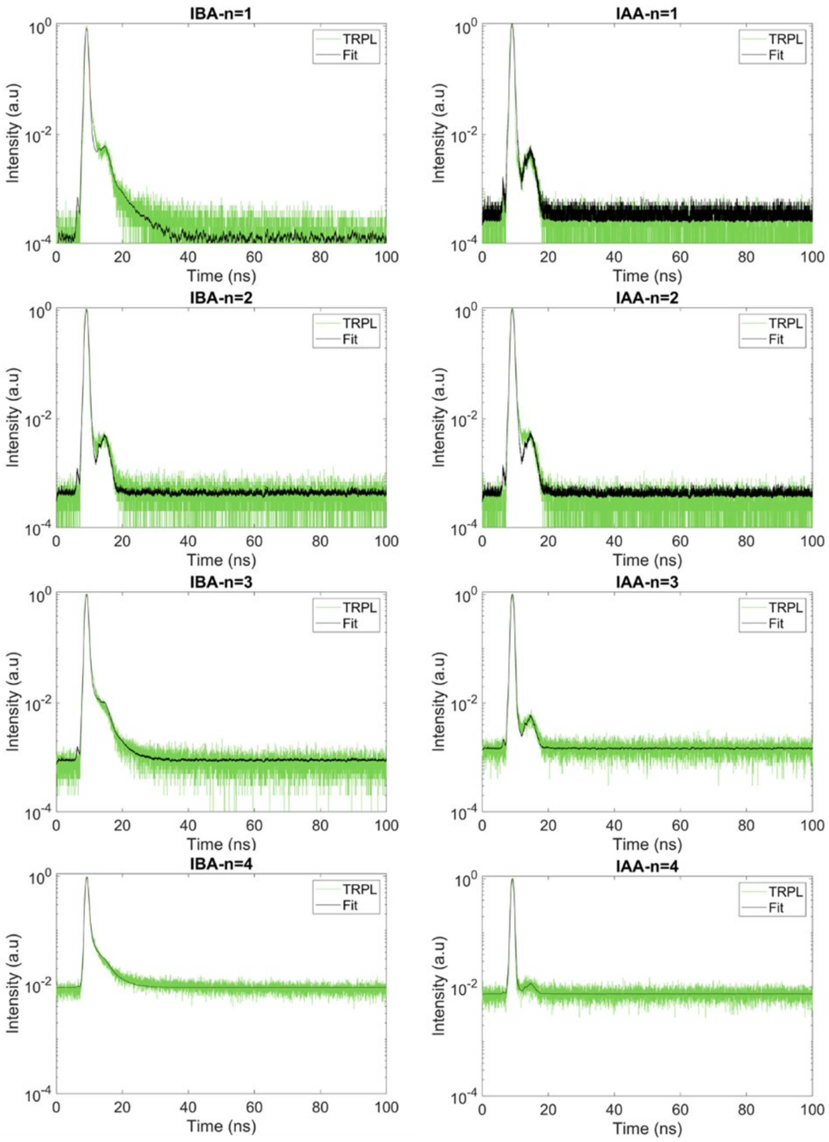

Figure S4. Time-resolved photoluminescence (TRPL) for both systems. In general, the lifetimes were too short for accurate measurements with our system.

Time-resolved photoluminescence (TRPL) was performed on the crystals, but the lifetimes were on the order of nanoseconds which was too short for our system to accurately measure. Surface effects or other defects may be prevalent in these systems, leading to lower lifetimes, but this is issue is beyond the scope of this study. This correlates to the reflectance data, which show long tails extending toward low energy, possibly indicating the presence of high-n members as surface impurities, which would quickly quench the dominant n phase.[6]

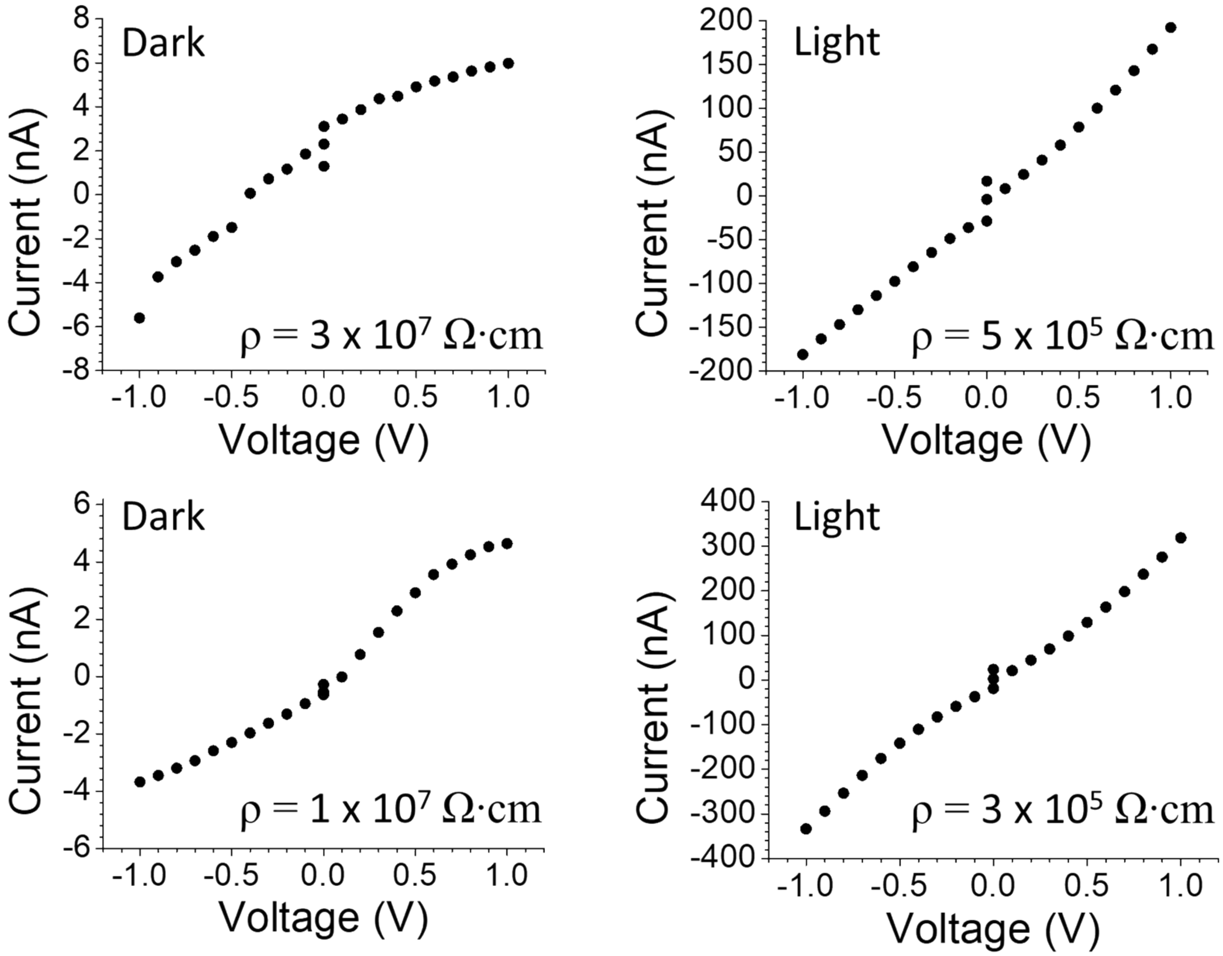

Figure S5. Resistivity measurements along the layers of the IBA (top) and IAA (bottom) n = 4 crystals. The decrease in resistivity under light is indicative of a photoresponse.

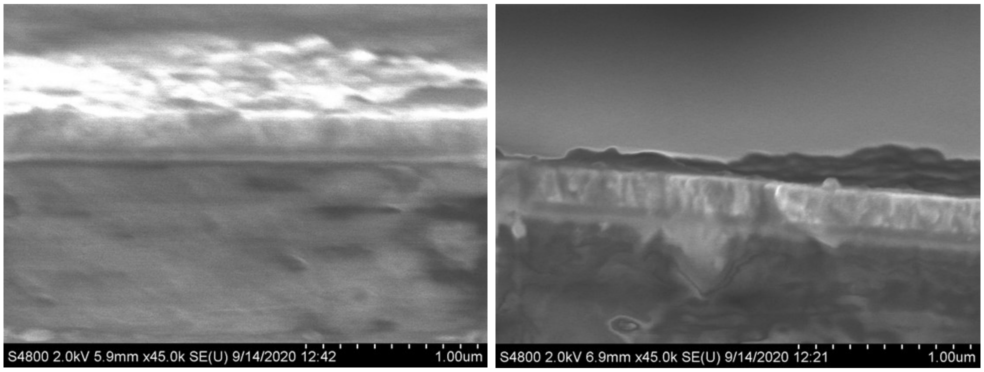

Figure S6. Cross-sectional scanning electron microscopy (SEM) images for films of IAA n = 4 (left) and IBA n = 4 (right) prepared with 2.5 wt% MACl added to the precursor solution on PEDOT:PSS/ITO/glass substrates.

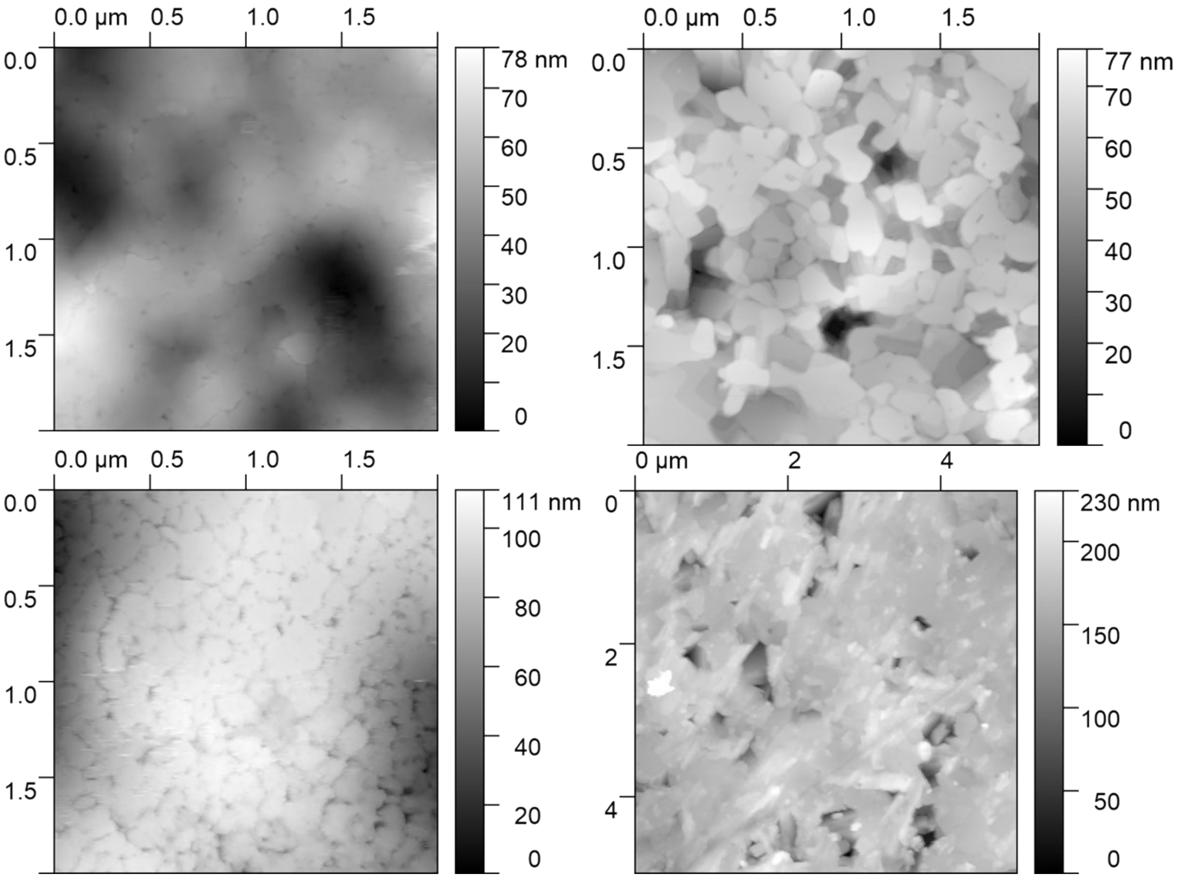

Figure S7. AFM of films of IAA (top) and IBA (bottom) without MACl (left) and with MACl (right).

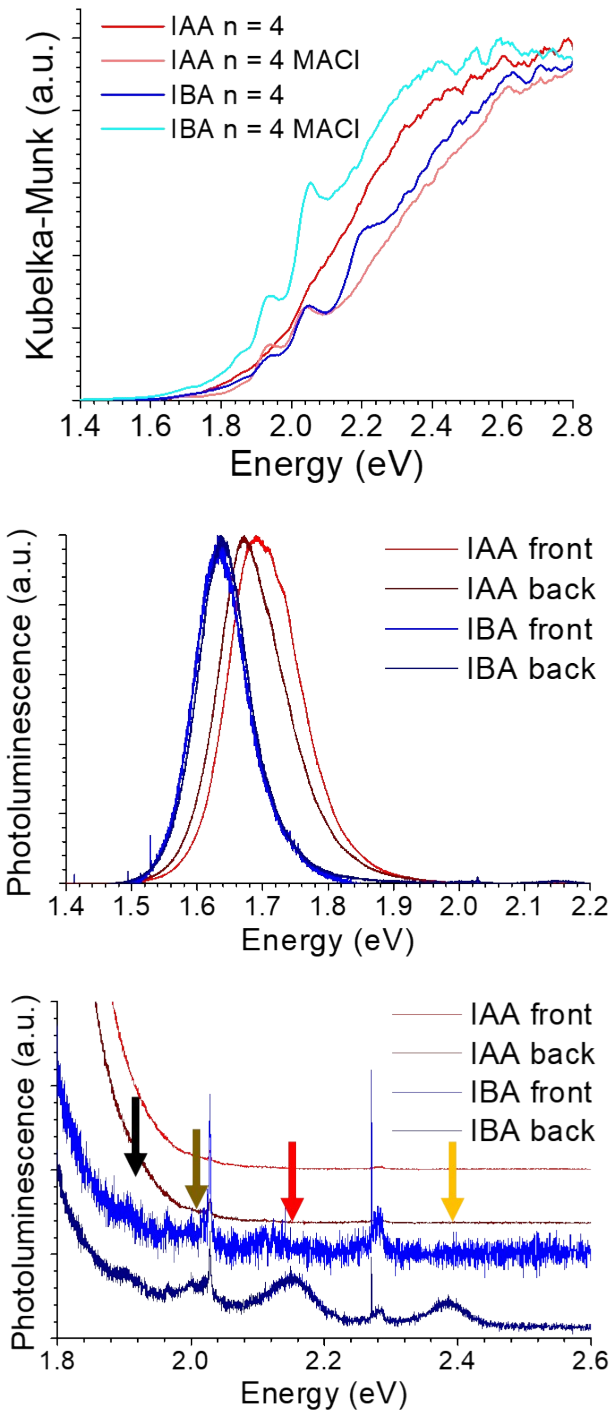

Figure S8. (top) The reflectance data, transformed by the Kubelka-Munk equation of films with and without an MACl additive. (middle) PL of the films with MACl taken from both the front and the back of the films. (bottom) Enlargement of the above PL graph emphasizing the low-n phases apparent from the back of the IBA film. Arrows highlight the various n values (1: yellow, 2: red, 3: brown, 4: black).

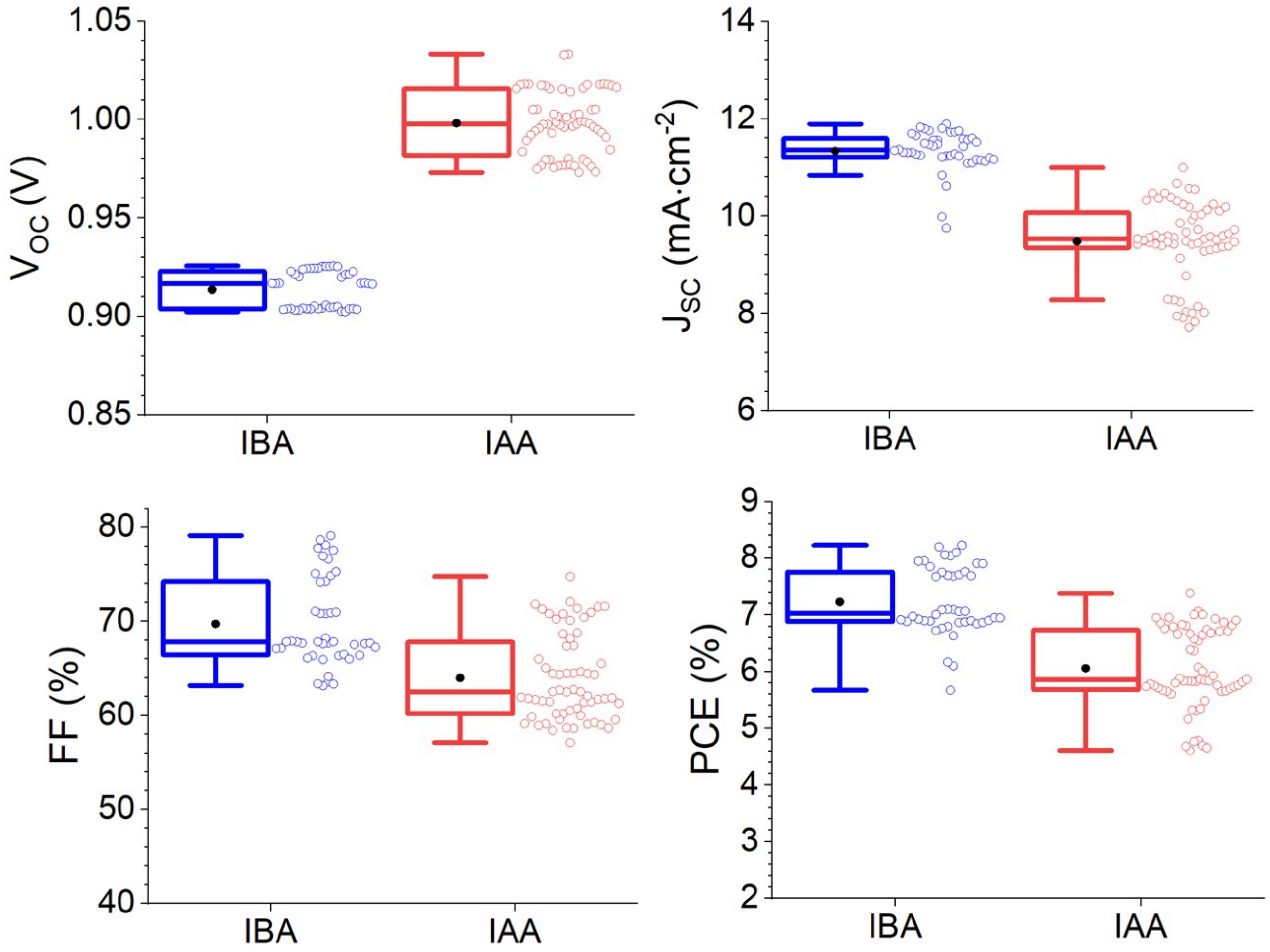

Figure S9. Statistics for solar cell devices using IBA and IAA n = 4 and a 2.5 wt% additive. For the box-and-whiskers plot, the black dot is the mean, the center line is the median, the box fills the middle two quartiles of data, and the whiskers cover 1.5 times the IQR. The data to the right are the data for each device made.

1. Petříček, V.; Dušek, M.; Palatinus, L., Crystallographic Computing System JANA2006: General features. *Zeitschrift für Kristallographie - Crystalline Materials* **2014,** *229* (5), 345.
2. Spek, A., Structure validation in chemical crystallography. *Acta Crystallographica Section D* **2009,** *65* (2), 148-155.
3. Kubelka, P.; Munk, F., Ein Beitrag zur Optik der Farbanstriche *Zeitschrift fur Technische Physik* **1931,** *12*, 593-601.
4. Slade, T. J.; Bailey, T. P.; Grovogui, J. A.; Hua, X.; Zhang, X.; Kuo, J. J.; Hadar, I.; Snyder, G. J.; Wolverton, C.; Dravid, V. P.; Uher, C.; Kanatzidis, M. G., High Thermoelectric Performance in PbSe–$NaSbSe_2$ Alloys from Valence Band Convergence and Low Thermal Conductivity. *Advanced Energy Materials* **2019,** *9* (30), 1901377.

5. Jiang, Z., GIXSGUI: a MATLAB toolbox for grazing-incidence X-ray scattering data visualization and reduction, and indexing of buried three-dimensional periodic nanostructured films. *Journal of Applied Crystallography* **2015,** *48* (3), 917-926.
6. Wang, N.; Cheng, L.; Ge, R.; Zhang, S.; Miao, Y.; Zou, W.; Yi, C.; Sun, Y.; Cao, Y.; Yang, R.; Wei, Y.; Guo, Q.; Ke, Y.; Yu, M.; Jin, Y.; Liu, Y.; Ding, Q.; Di, D.; Yang, L.; Xing, G.; Tian, H.; Jin, C.; Gao, F.; Friend, R. H.; Wang, J.; Huang, W., Perovskite light-emitting diodes based on solution-processed self-organized multiple quantum wells. *Nature Photonics* **2016,** *10*, 699.
